# AI-enhanced profiling of phage-display-identified anti-TIM3 and anti-TIGIT novel antibodies

**DOI:** 10.3389/fimmu.2025.1499810

**Published:** 2025-03-11

**Authors:** Astrid Musnier, Yannick Corde, Adrien Verdier, Mélanie Cortes, Jean-René Pallandre, Christophe Dumet, Adeline Bouard, AbdelRaouf Keskes, Zakaria Omahdi, Vincent Puard, Anne Poupon, Thomas Bourquard

**Affiliations:** ^1^ MAbSilico SAS, Tours, France; ^2^ Etablissement Français du Sang - Bourgogne Franche-Comté (EFS BFC), Plateforme ITAC-UMR1098-RIGHT, Besançon, France

**Keywords:** AI, affinity, developability, phage display, antibody, TIM3, TIGIT

## Abstract

Antibody discovery is a lengthy and labor-intensive process, requiring extensive laboratory work to ensure that an antibody demonstrates the appropriate efficacy, production, and safety characteristics necessary for its use as a therapeutic agent in human patients. Traditionally, this process begins with phage display or B-cells isolation campaigns, where affinity serves as the primary selection criterion. However, the initial leads identified through this approach lack sufficient characterization in terms of developability and epitope definition, which are typically performed at late stages. In this study, we present a pipeline that integrates early-stage phage display screening with AI-based characterization, enabling more informed decision-making throughout the selection process. Using immune checkpoints TIM3 and TIGIT as targets, we identified five initial leads exhibiting similar binding properties. Two of these leads were predicted to have poor developability profiles due to unfavorable surface physicochemical properties. Of the remaining three candidates, structural models of the complexes formed with their respective targets were generated for 2: T4 (against TIGIT) and 6E9 (against TIM3). The predicted epitopes allowed us to anticipate a competition with TIM3 and TIGIT binding partners, and to infer the antagonistic functions expected from these antibodies. This study lays the foundations of a multidimensional AI-driven selection of lead candidates derived from high throughput analysis.

## 1 Introduction

The new therapeutic strategy in clinical oncology which consists in activating the host’s immune system rather than killing the tumor itself, has laid the foundations for one of the greatest advances in recent medicine. These novel immunotherapies, known as ICI (immune checkpoint inhibitors), block the mechanisms that allow cancer cells to evade immune detection, and showed efficacy in many different cancer types (for review, see (1)). First approved in 2011 for targeting anti-cytotoxic T lymphocyte-associated protein 4 (anti-CTLA-4) in advanced melanoma, Ipilimumab was soon followed in 2014 by two anti-programmed cell death protein 1 (anti-PD-1) agents, Pembrolizumab and Nivolumab. Despite the tremendous improvement of patients survival, 10 years of clinical use drives to the conclusion that a majority of the patients are resistant to CTLA-4 and PD-1/PD-L1 axis immunotherapies (for review, see (1, 2)). Resistance to PD-1 blockade was demonstrated to originate from a complex interplay between the immune cells (CD8+ T cells, T regs, Myeloid-derived suppressor cells, Tumor-associated macrophages…) and the cancer cells within the tumor microenvironment (TME) (3–5). The release of immunosuppressive cytokines and negative cell interplays pushes CD8+ T cells toward exhaustion are the reported causes leading to the immune silencing of the TME. Exhausted T cells exhibit an increased expression of inhibitory immune checkpoints such as CTLA-4, PD-1, TIM3/HAVCR-2 (T-cell immunoglobulin mucin receptor 3/Hepatitis A virus cellular receptor 2), LAG3 (Lymphocyte activation gene 3), or TIGIT/VSIG9 (T-cell immunoreceptor with Ig and ITIM domains/V-set and immunoglobulin domain-containing protein 9). This accumulation is thought to be insurmountable for anti-PD-1 antibodies alone to restore T cells activation (6–8). Strategies are under clinical investigation to overcome PD-1 blockade resistance consisting, among others, in combining multispecific anti-ICI antibodies. Cooperation between TIGIT and PD-1 blockade (8), as well as between TIM3 and PD-1 blockade (7, 9), have previously been demonstrated efficient at overcoming T cell exhaustion. However, despite their promising potential to synergize with PD-1 and the numerous clinical trials initiated, no anti-TIGIT or anti-TIM3 are today FDA-approved and available for patients.

Therapeutic antibodies constitute the largest class of biologics, accounting for 140+ commercialized products, among which 5 reached the 10 top-selling drugs (10). Their tremendous success is mostly due to the high affinity and specificity they exhibit for their target, and subsequent limited (but not absent) side effects. As a counterpart, finding a therapy-level antibody is an obstacle race. The discovery phase is the first step of the antibody development, during which the lead antibodies are identified and optimized in order to fulfill the requirements of the preclinical and clinical trials. Many dimensions must be considered during the discovery: the ability to bind the target, on a functional epitope, with good affinity and high specificity, while eventually maintaining cross-species binding to anticipate the preclinical trials, and displaying good manufacturability properties. All these parameters allow to ensure that the antibody displays suitable production, safety and efficacy properties compatible with its use as a therapeutic molecule in human patients. Experimental antibody discovery forbids studying all these dimensions at the same time, the sequence of the wet-lab experiments being dictated by the number of molecules that can be handled by the state-of-the-art techniques. At the end, a complete antibody development, from the ignition of discovery to achieved clinical trials, takes an average of 7 years and costs over 1 b$. The massive amount of program failures (>95%) highlights the need for pipeline optimization.

*In silico* tools are being developed to rationalize antibody discovery, while limiting the number of experiments performed, late-stage failures and consequently the development costs of such molecules (11, 12). We developed several AI-based algorithms to predict antibody properties as early as when their sequences are known, such as epitope mapping, affinity prediction and developability assessment, among others (13–15). Here is presented a technological pipeline combining experimental phage display and AI-based epitope-driven affinity evaluation and developability assessment, which succeeded at identifying promising candidates against TIM3 and TIGIT.

## 2 Materials and methods

### 2.1 Cells lines, primary cells and culture

HEK293, CEM, SupT1 were purchased from ATCC (American Type Culture Collection, Manassas, VA). Jurkat and NK-92 were purchased from DMSZ (DSMZ-German Collection). NKL and YT were kindly provided by the ITAC platform-UMR1098 (France).

HEK293 cells were cultured in Dulbecco’s Modified Eagle medium (DMEM, Eurobio Scientific) supplemented with 10% fetal calf serum and 1% penicillin streptomycin (Eurobio). CEM, SupT1, Jurkat, JM, YT and NKL cell lines were cultured in Roswell Park Memorial Institute medium (RPMI 1640) supplemented with 10% fetal calf serum and 1% penicillin streptomycin (Gibco, France). NKL culture was supplemented with 200 UI/mL IL-2. NK-92 was cultured in alpha-MEM (with ribo- and deoxyribonucleoside) (PAN BIOTECH, Germany)) supplemented with 12,5% fetal calf serum, 12,5% horse serum, 200UI/mL of IL-2 and 1% penicillin streptomycin (Gibco, France). All cell lines were routinely tested for mycoplasma contamination.

Total blood was obtained from healthy donors using cytapheresis kits (French Blood Institute of Bourgogne Franche-Comté). Peripheral blood mononuclear cells (PBMC) were isolated by density gradient centrifugation (Ficoll-PaqueTM Premium, Dutscher, Brumath, France) and stained right away. Tissue-resident memory T cell (TRM) were obtained and cultured in RMPI supplemented with 10% fetal calf serum, 1% penicillin streptomycin, 50 ng/ml TGF-β1 (Peprotech réf 100-21) and 20 ng/ml IL-15 (Peprotech réf 200-15).

### 2.2 Targets genes design and synthesis

Genes were all obtained by gene synthesis (Twist Bioscience) and cloned in pcDNA3.1 via NEBuilder HiFi DNA Assembly. Transmembrane targets (TIM3: uniprot Q8TDQ0, TIGIT: uniprot Q495A1, SIRPα: uniprot P78324, PD-1: uniprot Q15116 and the Spike protein from Wuhan SARS-CoV-2: uniprot P0DTC2) were added by a Flag tag in C-term during design while TNFα (uniprot P01375) was added by a N-term Flag. The gene coding for the soluble RBD (Receptor Binding Domain, aa 335-528 of the Spike protein from Wuhan SARS-CoV-2, uniprot P0DTC2) was added by a signal peptide (aa from betaFSH gene, uniport P01225) in N-term to address to the secretion pathway and by to a linker containing a Flag Tag ((G3S)_2_-Flag Tag-(G3S)_2_) fused to the transmembrane domain of CD8 (aa183-235, uniport P01732) in C-term.

### 2.3 Phage display screening and primary validation of the scFv by ELISA

Phage display libraries screening was assessed on recombinant human His-tagged TIM3 or human His-Tagged TIGIT (ACROBiosystems) coated at 25 µg/ml for the first round, and 10 µg/ml for the second round on a Nunc MaxiSorp plates (Thermo Fisher). After blocking with 3% skimmed milk or BSA in PBS Tween, the bacteriophages were pre-cleared against a non-relevant His-tagged protein for 2h and the non-specific bacteriophages were eliminated by several washes with PBS Tween and PBS. At each round, the phages were eluted by trypsin digestion and were used to infect TG1 cells. From the second panning rounds on either TIM3 or TIGIT, 768 colonies were randomly picked, grown in 96 well format, and the scFv expression induced. Nunc MaxiSorp plates (Thermo Fisher) were coated overnight with 50 μl of 1 μg/ml of the target protein and the binding of scFv assessed by ELISA. Plates were washed thrice with PBS Tween and blocked with 200 μl of 2% BSA PBS Tween. After washes, 50 μL of unpurified scFv-containing bacterial culture supernatants in 2% BSA PBS Tween was added and left incubating at room temperature for 1 h. The plates were washed thrice with PBS Tween and 100 µl of Anti-HA tag antibody (Abcam) were added for 1h for scFv labelling. The plates were washed thrice with PBS Tween and 100 µl of HRP (horseradish peroxidase)-conjugated anti-Rabbit IgG (BioRad) was added. The Tetramethylbenzidine peroxidase (TMB) EIA substrate kit (Biorad) was used according to manufacturer’s instructions, and the absorbance was measured at 450 nm using a microplate reader (Biotek Instruments). Positive clones were individually sequenced (Azenta).

### 2.4 Antibody productions

The VH and VL genes of the phage-display isolated scFv were amplified by PCR (Q5^®^ high-fidelity, NEB Biolabs). The VH and VL genes of the control antibodies (anti-TIGIT antibodies Tiragolumab (PDB 8JEO, (16)) and MG1131 (PDB 7VYT, (17)), the anti-TIM3 antibodies M6903 (PDB 6TXZ, (18)), Tim3.18 (PDB 7KQL, (19)), Sabatolimab (20) and Cobolimab (21), the anti-SIRPα Fab CC-95251 (22), and the anti-SARS-CoV-2 Spike protein Casirivimab (23) and Bebtelovimab (24)) were obtained by gene synthesis (Twist Bioscience) and amplified. DNA amplicons were cloned using NEBuilder HiFi DNA Assembly into the pTrioz-hIgG1 (InvivoGen), a plasmid encoding the constant domains of human IgG1 (InvivoGen), and into an in-house variant of the pTRIOZ coding for murine IgG2a Fc fragment. HEK293 were then transiently transfected with purified plasmids using Metafectene (Biontex Laboratories GmbH) according to the manufacturer’s instructions. After 24 h, the medium was replaced by DMEM without red phenol and supernatants were harvested 48 to 72 h later. After concentration on Vivaspin Turbo 4 (3 kDa cut-off; Sartorius), IgG concentrations were evaluated with semi-quantitative ELISA, and/or quantitative HTRF (Homogeneous Time-Resolved Fluorescence) kit IgG HTRF Kappa MAb (Revvity) according to manufacturer’s instructions. Antibodies 6E9, 6TXZ and 7KQL were produced and purified under IgG1 format in a large batch by SinoBiological.

### 2.5 Binding assay by HTRF (homogeneous time-resolved fluorescence)

Buffers and donor or acceptor-coupled sensors were obtained from Revvity. Antibodies-containing HEK293 supernatants were diluted in PPI Terbium Detection buffer and the binding to TIM3 (6-His C-ter-tagged, Acro Biosystems) or TIGIT (TIT-H52H5 Human TIGIT Protein, His Tag, active dimer, AcroBiosystems) diluted at 0.6 ng/µl final concentration in small volume 384-wells plates (Greiner Bio-One). After 1h incubation, the anti-6His-d2 and anti-IgG1-Tb (Terbium) cryptate sensors were added according to manufacturer’s protocol, and the plates were incubated 1h in the dark. Fluorescence measurement was performed with a TriStar 2 LB 942 Multimode Microplate Reader (Berthold Technologies GmbH & Co) at 620 nm (donor background fluorescence) and 665 nm (binding signal). The HTRF ratio was calculated as the emission value at 665 nm divided by the 620 nm value, subtracted with reagent background ratio and multiplied by 10 000.

### 2.6 Flow cytometry-based binding assay

Binding to TIM3, TIGIT, SIRPα, PD-1 or RBD in HEK293 cells was performed on transiently-transfected cells using Metafectene (Biontex Laboratories GmbH) according to the manufacturer’s instructions. After 24 hours, the cells were trypsinized, fixed, and permeabilized according to the BD Bioscience CytoFix/CytoPerm kit’s protocol and distributed in 96-well plates at 50,000 cells per well. Cells pellets were resuspended in 50 µl of raw antibody supernatants (candidate antibodies or isotype) at room temperature for 1h. After one wash in 2 ml PBS supplemented with 2mM EDTA and 1% FBS, cells were co-incubated with 1 µg of APC-conjugated anti-human IgG antibody (Miltenyi Biotec) and 0.5 µg of PE-conjugated anti-Flag antibody (BioLegend) in 20 µl of Perm/Wash buffer at room temperature for 45 min. Cells were then washed with 100µl PBS-EDTA before suspension in 100 µl PBS-EDTA. Data (% of APC+PE+ cells and APC MFI of PE+ subpopulation) was collected with a MACSQuant Analyzer 10 (Miltenyi Biotec) and analyzed using FlowJo V10.

Prior to investigating the binding to endogenous TIM3 in CEM, SupT1, Jurkat, JM, YT, NKL and NK-92, a phenotypic analysis of the cell lines was realized in FACS buffer (PBS 1X, 0.2% BSA). TIM3 expression was evaluated with fluorophore-conjugated antibody F38-2E2 (Sony Biotechnology) and compared with an isotype. TIM3 positive lines were incubated with 1µg of 6E9 or 7KQL for 25 min in the dark at 4°C. After wash steps, the secondary detection was performed with PE-coupled anti-human IgG1 antibody (1/200e Jackson Immuno Research: 109-116-170) for 25 min in the dark at 4°C. For cell viability analyses, Fixable Viability Dye (eBioscience) was used according to the manufacturer’s instructions. Flow cytometry data was acquired on FACSymphony A1 (BD Biosciences) and analyzed with FlowJo software (v10.8.1).

PBMC were stained with an V450-coupled anti-CD3 antibody (BD Biosciences-clone OKT3) and a FITC-coupled anti-CD56 antibody (BD Biosciences-clone NCAM16.2). TRM (CD103+ CD69+ and CD8+) were stained with the previous antibodies and anti-CD103 (clone Ber-ACT8), anti-CD69 (clone FN50) and anti-CD8 (clone RPA-T8) antibodies (BD Biosciences).

### 2.7 Flow cytometry-based competition assays

HEK293 cells were transiently transfected with the target of interest and prepared as described above. Cells pellets were resuspended in 50 µl of raw hIgG1-formatted antibody supernatants (candidate antibodies or isotype) at room temperature for 1h. For antibodies pairwise competitions, 50 µl of mIgG2a-containing supernatants were added. For CD155 competitions on TIGIT, 1 µg of mIgG2a Fc-fused CD155 (ACROBiosystems) were added. Stainings of the remaining bound hIgG1 and the overexpressed targets, readings and data analyses are performed as described above.

### 2.8 K_D_ measurement by BLI (BioLayer interferometry)

All the measurements were performed with the Octet RED96 System (Pall Forte Bio) in PBS at 30°C and under 1000 rpm shaking. Anti-human Fc (AHC) biosensors (ForteBio) were soaked for 10 min in PBS, and saturated 15 sec in 1 mM biocytin. Sensors were rinsed twice in PBS for 120 sec and 60 sec, this step serving as background baseline. Sensors were loaded in antibody supernatants for 500 sec. Association with recombinant TIGIT or TIM3 proteins was done for 300 sec, and dissociation in PBS measured during 300 sec. Data was analyzed using Octet Software version 9.0. Experimental data was fitted with the binding equation describing a 1:1 interaction. Global analyses of the data sets assuming that binding was reversible (full dissociation) were carried out using the nonlinear least-squares fitting, allowing a single set of binding parameters to be obtained simultaneously for all concentrations used in each experiment.

### 2.9 Data analysis, statistics, graph edition and iconography

Statistical analyses and graphs were performed with GraphPad Prism 9 software. Pictures were built with biorender.com and thenounproject.com.

## 3 Results

### 3.1 Candidates identification and binding validations

**Figure 1:**
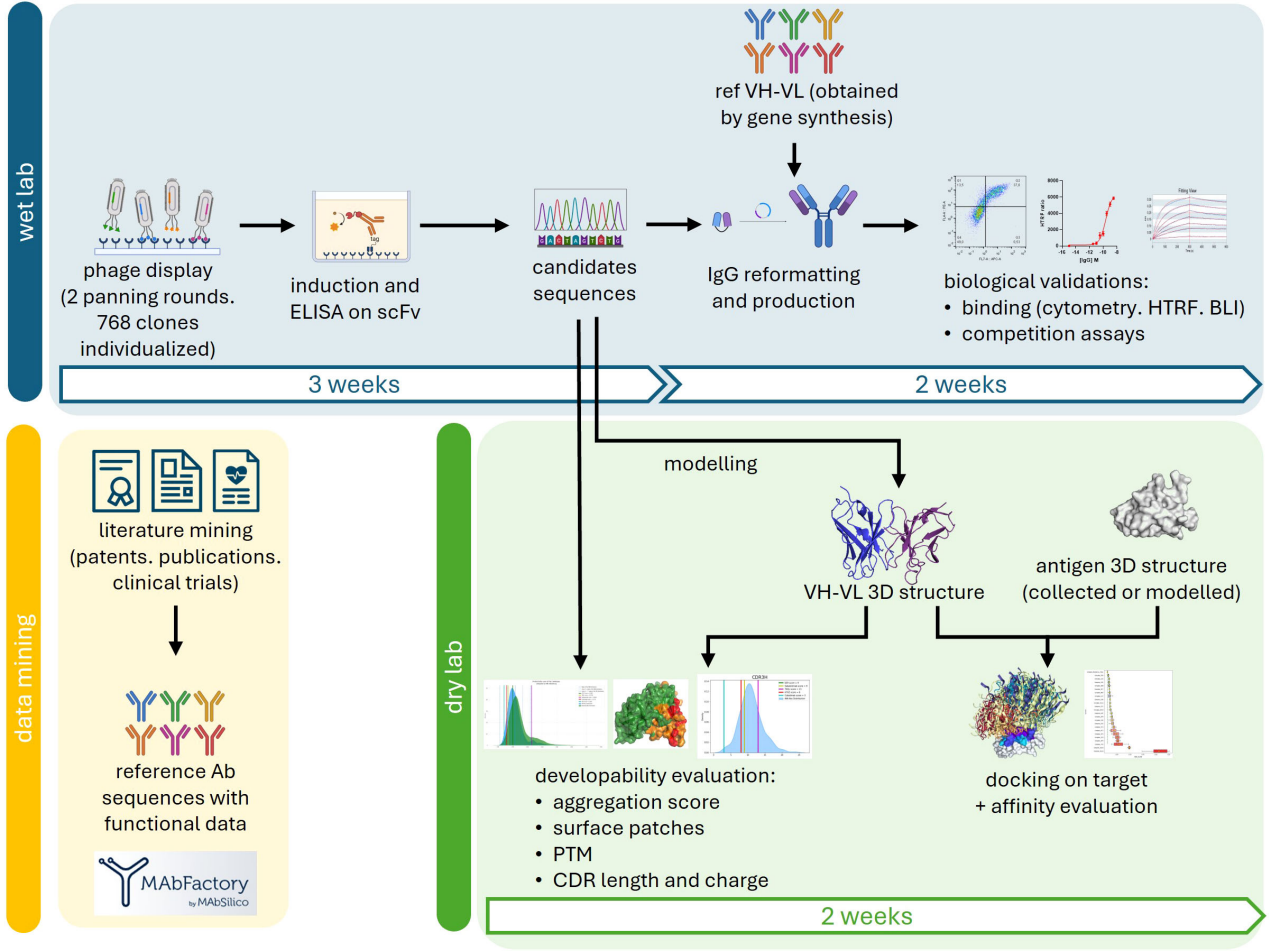
Global process of our approach. After conducting phage display screening and sequencing of the primary scFv candidates, further characterization were performed simultaneously in the wet lab (blue) and the dry lab (green). In the wet lab, the scFvs were reformatted into IgG, produced, and their binding was validated. Publicly available control data obtained through data mining (yellow) was used for comparison in the experiments and to evaluate the eligibility of the antibodies for intellectual property protection. In the dry lab, the 3D structures of the VH and VL domains were modeled through homology. Sequential and structural features of the antibodies were analyzed to assess their developability. The VH-VL structural models were docked onto the 3D structures of their respective targets, and the affinity of the resulting complexes was evaluated, allowing for the identification of the antibodies’ epitopes.

Phage display and hybridomas are efficient and robust methods to screen antibody libraries against a desired target. Both approaches are based on a common principle: to fish out the affinity outliers from the antibody collection. Important parameters of antibody discovery such as the developability potential of the identified hits or their epitope mapping are ignored during the selection despite their tremendous importance for the future of the candidates. The purpose of our study was to show the added value of our AI-based tools to provide developability and structural insights to a regular and fast phage display primary selection. The global process is described in **Figure 1**, and starts by identifying candidates and obtaining their sequences. A scFv-encoding phage display library was enriched along 2 rounds of panning against the recombinant extracellular domains of TIM3 or TIGIT and 768 clones of each pool were randomly picked. The scFv production was induced and an ELISA screening using a classical HRP-coupled anti-tag antibody identified 40 positive clones against TIM3 and 14 against TIGIT (data not shown). The VH and VL were sequenced and a primary analysis of sequence redundancy showed that all TIGIT clones and 25 out of the 40 TIM3 clones were unique. The 39 scFv candidates were reformatted as IgGs by fusing the VH and VL of the initial scFv to the constant domains of unmodified human IgG1. The IgG were directly screened from unpurified production supernatants by HTRF and flow cytometry to eliminate candidates displaying insufficient affinity, false positives, and the ones that lost their binding due to reformatting (**Supplementary Figure S1**). Publicly-disclosed reference antibodies directed at TIM3 (M6903 (PDB 6TXZ, (18)), Tim3.18 (PDB 7KQL, (19) and the Sabatolimab (20)) or TIGIT (Tiragolumab (PDB 8JEO, (16), and MG1131 (PDB 7VYT, (17)) were obtained by gene synthesis, cloned and produced in the same way and used as controls. Out of the 25 unique TIM3 clones, only 6E9 exhibited a binding during both assays after being reformatted (**Supplementary Figures S1A, C**). Among the 14 anti-TIGIT IgGs, 6 bound in HTRF to recombinant TIGIT, and 4 of those in flow cytometry: clones T2, T4, T7 and T10 (**Supplementary Figures S1B, D**).

**Figure 2:**
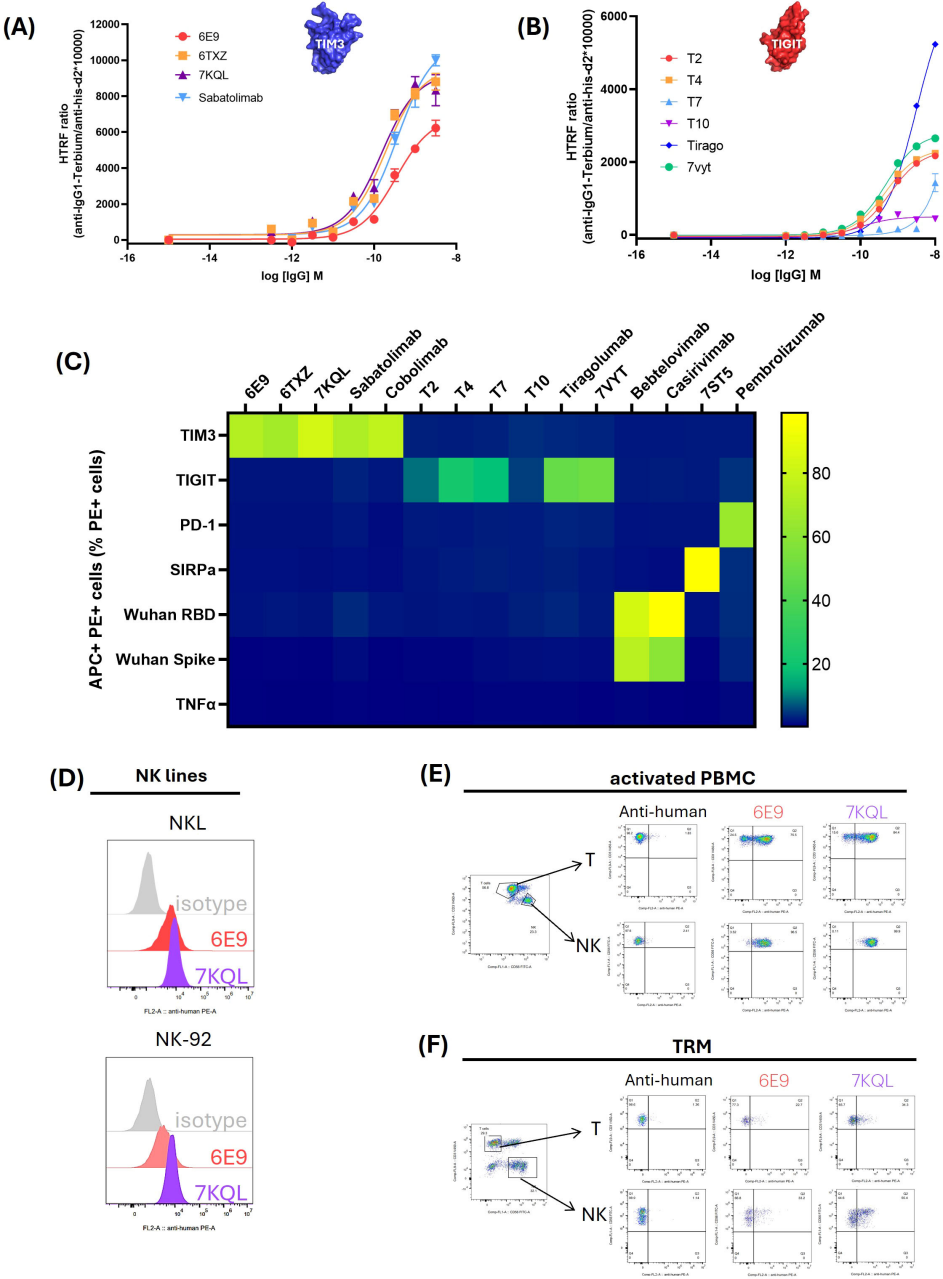
Binding validation and specificity assessment of the 5 final candidates. The 5 final candidates and the reference antibodies were produced as human IgG1 in HEK293 supernatant, concentrated and dosed. **(A, B)**: Binding validation. For HTRF assays, the recombinant extracellular domains of TIM3 **(A)** or TIGIT **(B)** fused to a biotinylated Avitag were incubated with increasing concentrations of the antibodies. Antibodies and targets were detected with fluorophore-coupled sensors: d2 acceptor coupled to an anti-IgG and terbium donor coupled to the streptavidin. The binding was assessed as the energy transfer between the donor and acceptor and computed as the HTRF ratio: 665nm acceptor emission/620 nm donor emission x 10,000. Curves were fitted mathematically with GraphPad Prism software. **(C)** Specificity evaluation of the candidates. HEK293 cells were transiently transfected with the Flag-tagged target gene and incubated with the candidate or reference antibodies. The target expression was monitored with a PE-coupled anti-Flag antibody and the binding of the antibodies was followed with an APC-coupled anti-IgG. Percentage of APC+ PE+ cells among the total PE+ cell population was indicated through a color gradient. **(D–F)** Binding of endogenous TIM3 by 6E9 in immune-relevant cells. NK lines NKL and NK-92 **(D)**, activated PBMC **(E)** and TRM cultured with TGF-β1 and IL-15 **(F)** were incubated with 6E9 or 7KQL as a control. The T and NK lymphocytes subsets of the PBMC and TRM were optically isolated after staining of CD3 and CD56 (CD3+ T lymphocytes and CD3-CD56+ NK lymphocytes).

The 5 final candidates were produced at larger scale, quantified and moved to more precise *in vitro* characterization. The HTRF ratios were measured in dose-response in order to obtain comparable EC50s. 6E9, T2 and T4 displayed similar profiles as compared to reference antibodies, with EC50 in the sub-nM range (**Figures 2A, B**; **Table 1**). T7 and T10 exhibited delayed binding in this assay. K_on_, K_dis_, and K_D_ measurements were performed by BLI in order to obtain kinetic insights into the binding (**Table 1**; **Supplementary Figure S2**). 6E9 exhibits association and dissociation profiles similar to the references 7KQL and Sabatolimab, and consequently a similar K_D_. The 6TXZ displayed a slightly better affinity in virtue of a better K_dis_. T4 displayed the best binding properties among the TIGIT candidates because of a slightly better K_dis_.

**Table 1:** Binding constants.

Candidates		HTRF	BLI		
		EC50 (M)	KD (M)	kon(1/Ms)	kdis(1/s)
anti-TIM3	6E9	3.422E-10	1.30E-09	4.29E+05	5.56E-04
	7KQL	1.499E-10	1.01E-09	2.16E+05	2.19E-04
	6TXZ	1.931E-10	2.00E-10	1.53E+05	3.06E-05
	Sabatolimab	3.638E-10	2.44E-09	1.57E+05	3.82E-04
anti-TIGIT	T2	6.23E-10	3.19E-09	4.50E+05	1.44E-03
	T4	4.65E-10	2.00E-09	4.48E+05	8.96E-04
	T7	~ 1.796e-005	2.53E-09	6.37E+05	1.61E-03
	T10	9.749E-11	3.07E-09	4.96E+05	1.52E-03
	7VYT	4.885E-10	1.89E-10	3.93E+05	7.43E-05
	Tiragolumab	3.145E-09	6.81E-10	4.10E+05	2.79E-04

EC50 were obtained from mathematical fit of the HTRF binding data. K_D_, K_on_ and K_dis_ were obtained by BLI.

The binding specificities of the antibodies were assessed by flow cytometry in HEK293 cells overexpressing selected targets. PD-1 and SIRP-α, which belong to the V-set structural family like TIGIT and TIM3, as well as the non-related Spike protein (Wuhan SARS-CoV-2), RBD (Receptor Binding Domain of the Wuhan SARS-CoV-2 Spike protein) and TNFα, were investigated (**Figure 2C**). All the targets were fused to a Flag tag which allowed to optically select the target-overexpressing subpopulation (PE+ staining), and the binding of the antibodies was detected with an APC-coupled anti-IgG antibody. The percentage of APC+PE+ cells were collected and reported in the matrix (the cytometry plots are shown in **Supplementary Figure S3**). As can be observed, the identified anti-TIGIT and TIM3 candidates bound their cognate target, but neither to other V-set proteins, nor to the non-related RBD, Spike or TNFα. As expected, the investigated targets bound their known control antibodies: Pembrolizumab on PD-1, 7ST5 (Fab CC-95251) on SIRP-α, and Bebtelovimab and Casirivimab on the RBD and the trimeric Spike.

The antibodies aim at being used as immune checkpoint inhibitors to restore T cell function in the tumor microenvironment (TME). Therefore, 6E9 binding was investigated in TME-relevant T-cell models. The endogenous expression of TIM3 was first evaluated by flow cytometry in four T cells lines (CEM, JM, Jurkat and SupT1), three NK lines (NK-92, NKL and YT), and human primary PBMC (peripheral blood mononuclear cells) and TRM (tissue-resident memory T cell). Detectable levels of TIM3 were observed in NK-92 and NKL lines, as well as in both the CD3+ (T) and CD3-CD56+ (NK) subsets of PBMC and TRM (**Supplementary Figure S4**). Interestingly, whereas TIM3 was not detected in T lines as described in the literature, its expression was observed in primary PBMC and TRM subsets. The binding of 6E9 and its control 7KQL were consistently observed on TIM3-expressing cells, opening avenues for future *in vitro* and *in vivo* studies (**Figures 2D–F**).

We herein identified one anti-TIM3 and 5 anti-TIGIT antibodies, which all seemed to display good binding properties and good specificity.

### 3.2 Developability assessment

Throughout the antibody development process, it is crucial to ensure that its physical and chemical attributes will ensure the patient’s safety, while achieving the desired pharmacokinetic profile, and maximizing therapeutic effectiveness. Assessing the developability requires evaluating balancing factors such as the production yield, protein heterogeneity, aggregation rate, viscosity, and immunogenicity, to ultimately evaluate a probability of success along the path from discovery to development. Usually evaluated very late in the process with experimental methods, these parameters are not considered as selection criteria during early stages. Our computational developability assessment tool analyzes antibody sequences and structures, comparing them with publicly available antibodies to demonstrate favorable developability traits very early in the process, as soon as the selection is done.

#### 3.2.1 Patent mining, sequence identity analysis, and intellectual property evaluation

Both functional and sequential novelties are necessary to establish an antibody’s eligibility for intellectual property protection. However, determining the threshold for comparing two antibodies remains unclear. An arbitrary sequence identity threshold of 80% was set, corresponding to 50 amino acid differences between two full-length VH-VL pairs, and 10 amino acid differences across two sets of six CDRs, to define the sequential uniqueness of antibodies.

We collected a unique database of manually curated monoclonal antibodies sequences from patents which are accompanied by, when disclosed, functional data like epitope residues, affinity, cross-species reactivity and functional studies (https://app-publicdemo-mabfactory-97288.azurewebsites.net/). To date (Aug 2024), our database references 87,592 antibodies directed at 3,046 targets, and associated affinity data for 21,241 of them, epitope data for 9,564, cross-specificity data for 3,777, and EC50/IC50 from functional assays for 10,411. At the time of writing this article, our database referenced 170 anti-TIM3 antibodies and 1004 anti-TIGIT antibodies with which we compared our 5 candidates (**Table 2**). 6E9 did not exceed 79% of sequence identity with any of the anti-TIM3 antibodies reported in our database. Moreover, the closest antibody, Pab2088 from patent US20170368168A1, displayed no more than 52% sequence homology when considering the 6CDRs only. The anti-TIGIT candidates T2, T4 and T7 exhibited 87, 86 and 85% full-length sequence identities respectively with antibodies described in the patent US10759855B2 (Ab33, 26 and 114 respectively). Interestingly, when focusing on the 6CDRs, the sequence identity with Ab33, 26 and 144 did not exceed 69%. When searching for the closest antibodies focusing only on the 6 CDRs of T2, T4 and T7, different antibodies (but still from patent US10759855B2) were retrieved from the database: Ab45 for T2, Ab44 for T4, and Ab 26 and 36, for T7. Their similarity capped at 71%. Finally, T10 was found close to the Ab clone 18 from patent WO2018160704A1, with which it displayed 89% sequence identity at the full-length level, but only 74% when considering only the 6 CDRs.

**Table 2:** Maximal sequence identities with the reference database.

Candidates	Full-length max seq id		6CDRs max seq id	
	Seq id	Database Ab	Seq id	Database Ab
T2	0.87	Ab33. patent US10759855B2	0.71	Ab45. patent US10759855B2
T4	0.86	Ab26. patent US10759855B2	0.69	Ab44. patent US10759855B2
T7	0.85	Ab114. patent US10759855B2	0.58	Ab26 & Ab36. patent US10759855B2
T10	0.89	clone 18. patent WO2018160704A1	0.74	clone 18. patent WO2018160704A1
6E9	0.79	Pab2088. patent US20170368168A1	0.52	Pab2088. patent US20170368168A1

The sequences of our 5 candidates were confronted with 170 anti-TIM3 antibodies and 1004 anti-TIGIT antibodies collected in our internal database. The table shows the antibodies displaying the maximal sequence identity with our candidates, on the full-length sequences or restricted to the 6 CDRs only. Their names and initial patents are reported.

Altogether, these results showed that the anti-TIM3 and anti-TIGIT leads displayed satisfying sequence distance with the publicly disclosed antibodies, supporting the prospect of securing full intellectual property protection.

#### 3.2.2 Germline drift and PTM motifs analysis

Naturally occurring somatic hypermutation drifts the antibody from its germline, and introduces modifications in the sequence and global surface of the VH/VL pair (25). Sequential motifs can appear on which the antibody may undergo chemical modifications known as post-translational modifications (PTM). They can happen during manufacturing, storage or even in the patient’s blood stream and can ultimately alter the antibodies potency, efficacy, and safety (for review, see (26)). The most commonly investigated PTM sites englobe: N-glycosylations (N-X-S/T, N-X-C), lysine glycations (K-D/E), asparagine deamidation (N-G/S/D/T/H), aspartate isomerization (D-G/S/N/T/H), aspartate-proline cleavage, methionine and tryptophan oxidation, and cysteine hydrolysis (27–31). We developed our own tool to investigate PTM motifs in the antibody sequences. Importantly, it has to be remembered that no predictive method provides a straightforward proof that the identified PTM-prone residues are actually going to be modified, nor that they are going to be detrimental for the antibody’s function. In order to fully evaluate the risk associated with an identified motif, evolutionary and structural layers of analysis were added in our method. First, considering that the developability risk associated with a PTM is lower when the PTM motif is also present in the closest germline, we combined germline analysis and PTM motifs search. Second, since PTMs happen after protein translation and folding, they are less (or not) likely to occur on residues that are not exposed at the surface of the antibody. We hence modeled the 3D structures of the antibodies and evaluated the solvent exposure of the PTM-prone residues.

**Figure 3:**
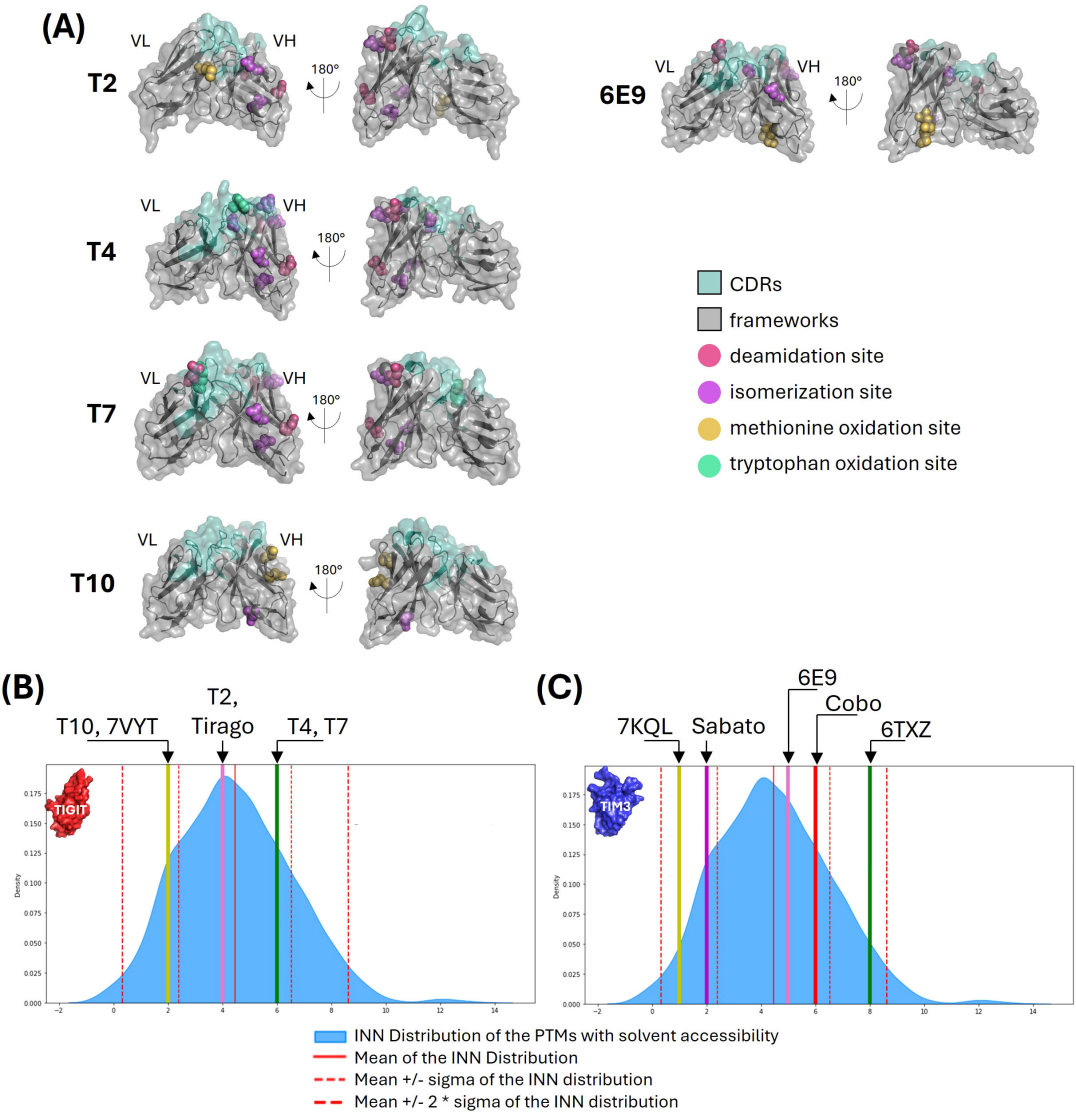
Post-translation modifications analysis. **(A)** PTM motifs shown on VH-VL 3D representations. The main PTM-prone residues of the identified deamidation, isomerization, and methionine or tryptophan oxidation sites are shown in colored spheres. The antibodies surfaces are shown in grey (frameworks) and pale green (CDRs). **(B, C)** Comparison with INN antibodies. The number of PTM motifs with solvent accessibility higher than 30% were computed from 735 INN antibodies. The number of motifs identified for our anti-TIGIT **(B)** and anti-TIM3 **(C)** candidates and their references are shown as colored lines are compared with the distribution of the INN antibodies shown in blue.

All five antibodies had unique germline combinations (data not shown). The VH fragments from T4 and T7 were the only to show no mutation from their respective germlines, the other ones having between 3 and 13 mutations. PTMs motifs were identified in each variable fragment, 21% displaying low exposure (< 30% of their lateral chain surface), 51% displaying medium exposure (31 to 60%) and 28% displaying high exposure (> 61%). **Figure 3A** shows the PTM-prone residues identified, except for the putative lysine glycation sites which were too numerous. No glycosylation site was detected. A vast majority of the PTM motifs were conserved from the germline and located in the frameworks (over 86%). T4, T7 and 6E9 exhibited CDR-located PTM motifs, among which two were highly exposed: an isomerization site in T4 CDRH2, and a deamidation site in T7 CDRL1. The number of PTM motifs with solvent accessibility higher than 30% were extracted and compared with the distributions computed from 735 INN (International Nonproprietary Names) antibodies (**Figures 3B, C**). These latter ones reached the clinical trials and were therefore considered to display good developability properties. T4 and T7 displayed the most numerous exposed motifs (6), and T10 the least (2). However, they all favorably compared to INN antibodies and no PTM-associated risk was clearly identified.

Overall, the five antibodies exhibited favorable profiles regarding the number of PTMs and their solvent accessibility. However, attention should be maintained on T4 and T7 due to the motifs located within their CDRs.

#### 3.2.3 Aggregation and immunogenicity risks evaluation

**Figure 4:**
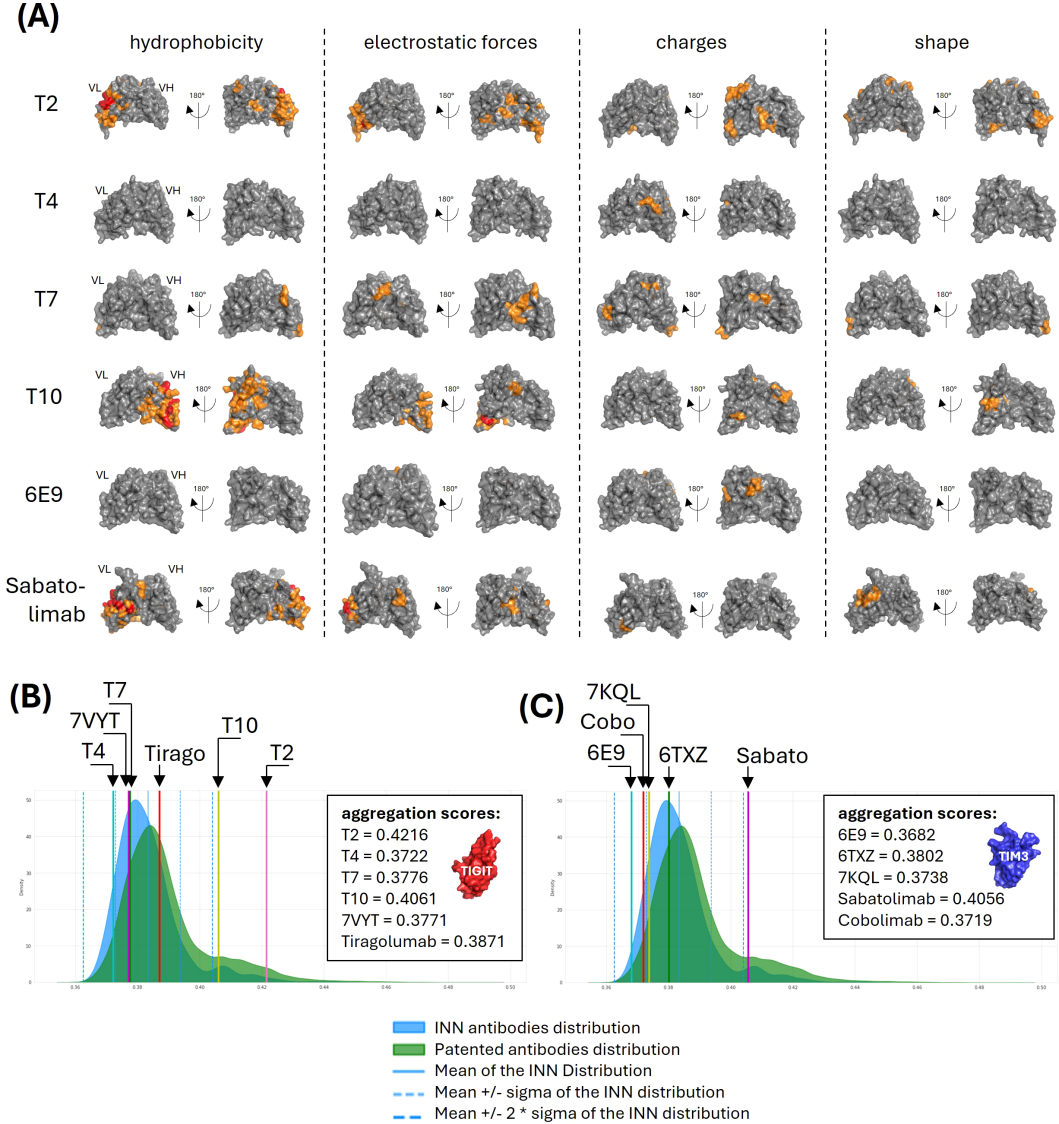
Aggregation parameters analysis. **(A)** Surface physicochemical patches. Surface properties (i.e. hydrophobicity, electrostatic forces, charges and shape) of the VH-VL pairs were computed and moderate (orange) to intense (red) regions were shown on the VH-VL structures. **(B, C)** Aggregation scores comparison with publicly-disclosed antibodies. The surface properties were used to train a model allowing to predict an aggregation score. Scores were computed for our anti-TIGIT **(B)** and anti-TIM3 **(C)** candidates and their references, and compared with the scores distribution computed from 735 INN antibodies (blue) and 31,712 patented antibodies (green).

The solubility-aggregation balance of a protein preparation depends on both the solution conditions (e.g., temperature, pH, ionic strength, excipients) and the intrinsic properties of the protein (e.g., physicochemical and structural characteristics). Typically, self-aggregation (colloidal instability), hydrophobicity, and local structural instabilities are analyzed using chromatographic and spectroscopic techniques, while formulation optimization is often employed to reduce aggregation. Several computational approaches have been developed to predict aggregation propensity and propose corrective measures (for a review, see (12)). We developed a structure-based tool to evaluate the surface physicochemical properties of antibodies. The VH and VL domains of the antibody were modeled using a rigid, coarse-grained representation, and the surface was divided into N centroids, where N can vary between 50 and 250. For each centroid, hydrophobicity, electrostatic forces, charges, and local curvature were calculated, enabling the identification of surface regions with similar properties. **Figure 4A** highlights the most problematic regions found for our candidates. As can be observed, T4 and 6E9 displayed less problematic regions across the surface, with only slightly charged residues identified in their VH fragments. T7 displayed an intermediate profile, with very localized small regions on its VL considering the four investigated properties. The most problematic were T2 and T10 which displayed large and intense hydrophobic patches in their VL and VH, respectively. T2 moreover exhibited patches of high electrostatic forces (VL) and high charges (VH).

An isolation forest model was trained on all antibody structures available in the Protein Data Bank. This approach was chosen for its effectiveness in anomaly and outlier detection, with the aim of identifying candidates which physicochemical and geometrical properties deviate significantly from the majority of known antibodies. The five antibodies were processed according to the same computation and their scores compared with the controls distributions (**Figures 4B, C**). Consistently with their bad hydrophobic properties, T2 and T10 displayed the worst aggregation scores, with values of 0.4216 and 0.4061, placing them more than 2 standard deviations away from the INN distribution mean, indicating potential solubility issues. Interestingly, the Sabatolimab also scored outside of the INN mean ± 2sd consistently with a large hydrophobic region, a strong electrostatic region and an abnormally convex region identified on its VL (**Figure 4A**, lower panels). All the other antibodies, as well as the references, displayed a satisfying aggregation score.

Since the constant parts of the IgG are globally conserved in sequence and structure, the highly diverse CDRs are often thought to be responsible for the variability of aggregation, viscosity, and immunogenicity profiles. Abnormally long CDRs may constitute a risk in regard to aggregation either because the larger distance to the closest germline might correlate with increased immunogenicity, or because extended CDR might introduce conformational flexibility and subsequently local or global misfolding of the antibody. Longer CDRs also increase the risk of exposing hydrophobic residues and creating local secondary structures prone to aggregation (like β-sheets). The CDR charges was also demonstrated to have an impact on developability properties. Negatively charged CDRs were correlated with lower aggregation and higher viscosity (32–34), while positively charged CDRs were correlated with increased off-target recognition and specificity decrease (35). The CDRs lengths and charges were computed for the 735 INN antibodies, and our candidates compared to this distribution. No discrepancy of either length or charges was observed (**Supplementary Figure S5**).

**Figure 5:**
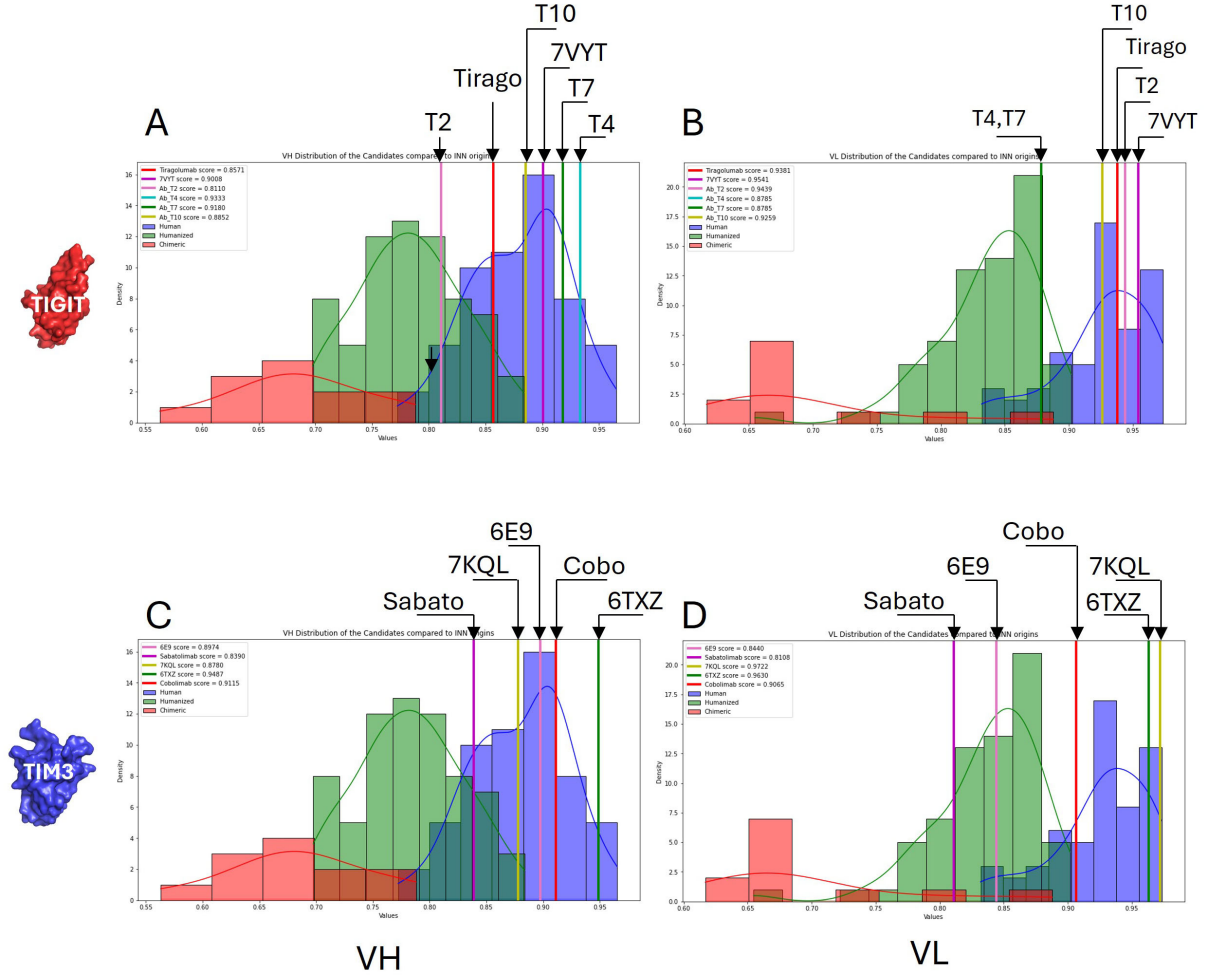
Humanness evaluation. A humanness score was computed from publicly available antibody sequences which allowed discrimination between human, humanized and non-human sequences (from chimeric antibodies). Separate scores were computed for the VH **(A, C)** and the VL **(B, D)**. The scores obtained for our anti-TIGIT (upper panels) and anti-TIM3 (lower panels) candidates are compared with the benchmark distributions.

Injecting high doses of monoclonal antibodies in the body may induce an immunogenic reaction. However, immunogenicity is thought to be limited when the antibody sequence remains close to a human one (36). From the antibodies sequences collected in our database, we computed a score to evaluate the humanness of the VH and VL fragments. As can be seen in **Figure 5**, our humanness score discriminates well the subsets of human, humanized or chimeric (non human) antibodies. All the tested antibodies were located in the human and humanized regions of the distribution, which is a good indicator of low immunogenicity. The worst score was displayed by Sabatolimab’s VL.

#### 3.2.4 Off-target effect prediction

**Figure 6:**
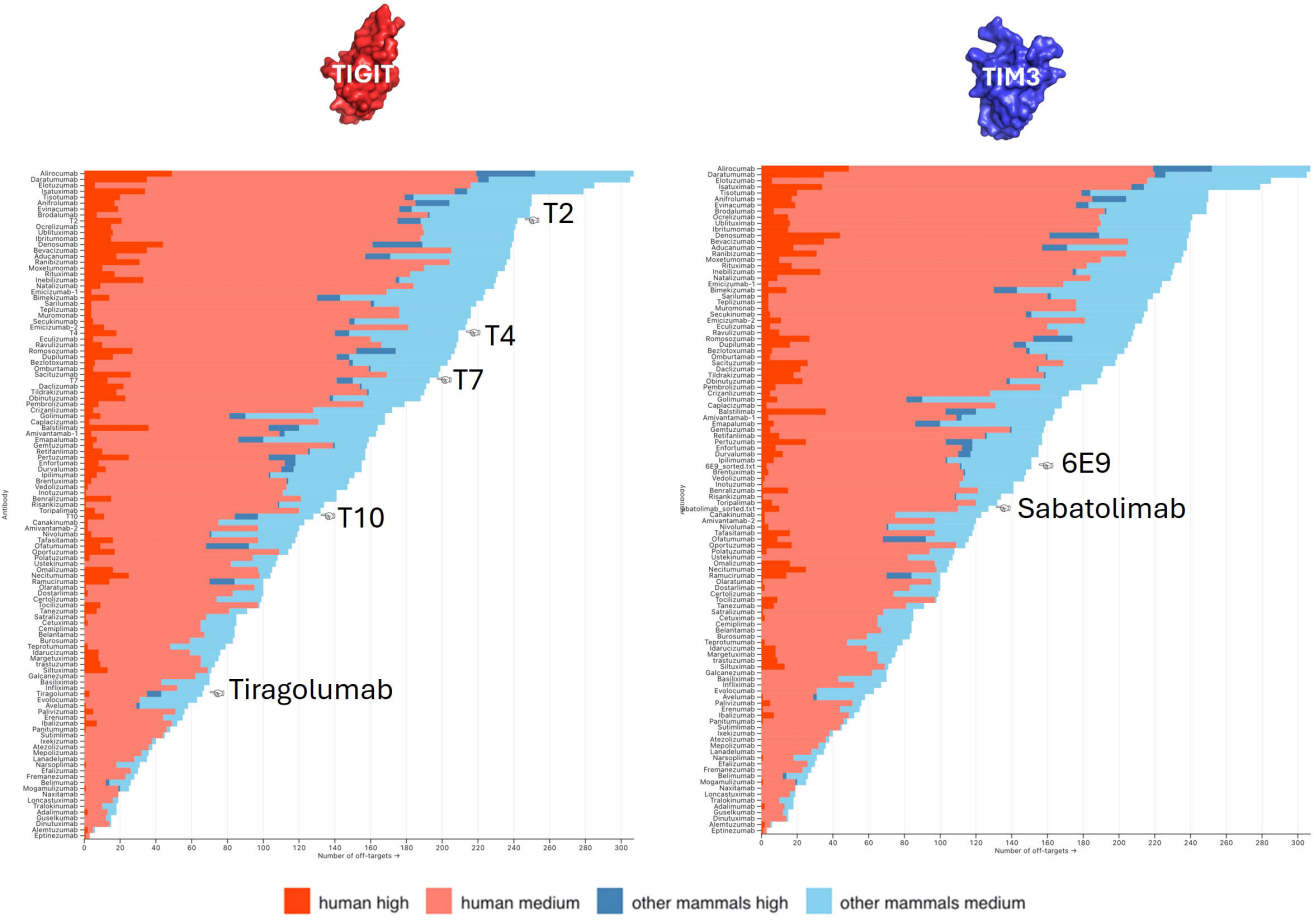
Off-targets prediction. The numbers of predicted off-targets, either human (red) or non-human (blue), were computed for all FDA-approved antibodies and ranked as a probability of being recognized (high vs. medium). The number of off-targets predicted for our anti-TIGIT (left panel) and anti-TIM3 (right panel) candidates were compared with that scale.

Antibody polyspecificity, defined as the capability of an antibody to bind with significant affinity to structurally and/or functionally different targets, has long been an understudied aspect of therapeutic antibody development. This is majorly due to the common belief that antibodies are mono-specific and that, at worst, may bind only close homologs of their targets. Polyspecificity was related to increased clearance and decreased PK (pharmaco-kinetics) characteristics (37). But more concerning, off-target recognition and subsequent off-site toxicities are shown to be responsible for failure during clinical development (38–40). Experimental methods to assess polyspecificity remain tedious and lack exhaustiveness. We previously developed MAbCross, a method to predict antibody off-targets based on CDR similarity measurement (13, 15). The predicted off-targets are ranked according to a score representing the binding probability and according to the species of the protein (human vs. non-human). For comparison purposes, the number of off-targets were calculated for FDA-approved antibodies and a scale was drawn on which the candidates are reported (**Figure 6**). 6E9 was by far the one displaying the lowest risk of human off-target binding, with only 4 proteins detected at a maximal score of 54.25/100. T2 and T4 were characterized as the riskiest, with 23 and 19 high probability off-targets, the top being scored over 80/100. T7 and T10 displayed medium risk.

### 3.3 Epitope characterization, affinity prediction and validation of the structural model

One of the major drawbacks of phage display or hybridoma-based discovery processes is the tedious definition of the epitope and the obtention of the structural model of the antibody-antigen pair. Epitope mapping is typically performed with low throughput technologies (X-ray, NMR, HDX-MS etc) at late stages of antibody discovery, on a limited number of candidates. In the early stages, competition assays with either the ligand or other antibodies can suggest overlapping binding sites and provide an indication of the binding region. However, they do not reveal the precise epitope. We previously developed MAbTope, a docking-based epitope mapping method (14, 41). MAbTope uses coarse grain representation of the antibody and the target to deduce the most favorable epitope region from AI-ranked docking poses. Among these poses, it is probable that a close-to-crystal one exists, but the method does not allow to identify which one. We hence ambitioned to develop an affinity prediction method which would allow us to evaluate the interaction of oriented docking poses and hence decipher the most accurate structural model of the antibody-antigen complex. Our affinity method uses coarse-grained representation of epitope and paratope (42) and computes a combination of parameters of the antibody-antigen interface: i/the physico-chemical complementary (PCC) on the facing regions computed as the PCC score, ii/the interacting complementarity score defined as the number of interacting regions between the paratope and epitope computed as the IR-score, and iii/a combination of the 2 parameters computed as the final C-score.

**Figure 7:**
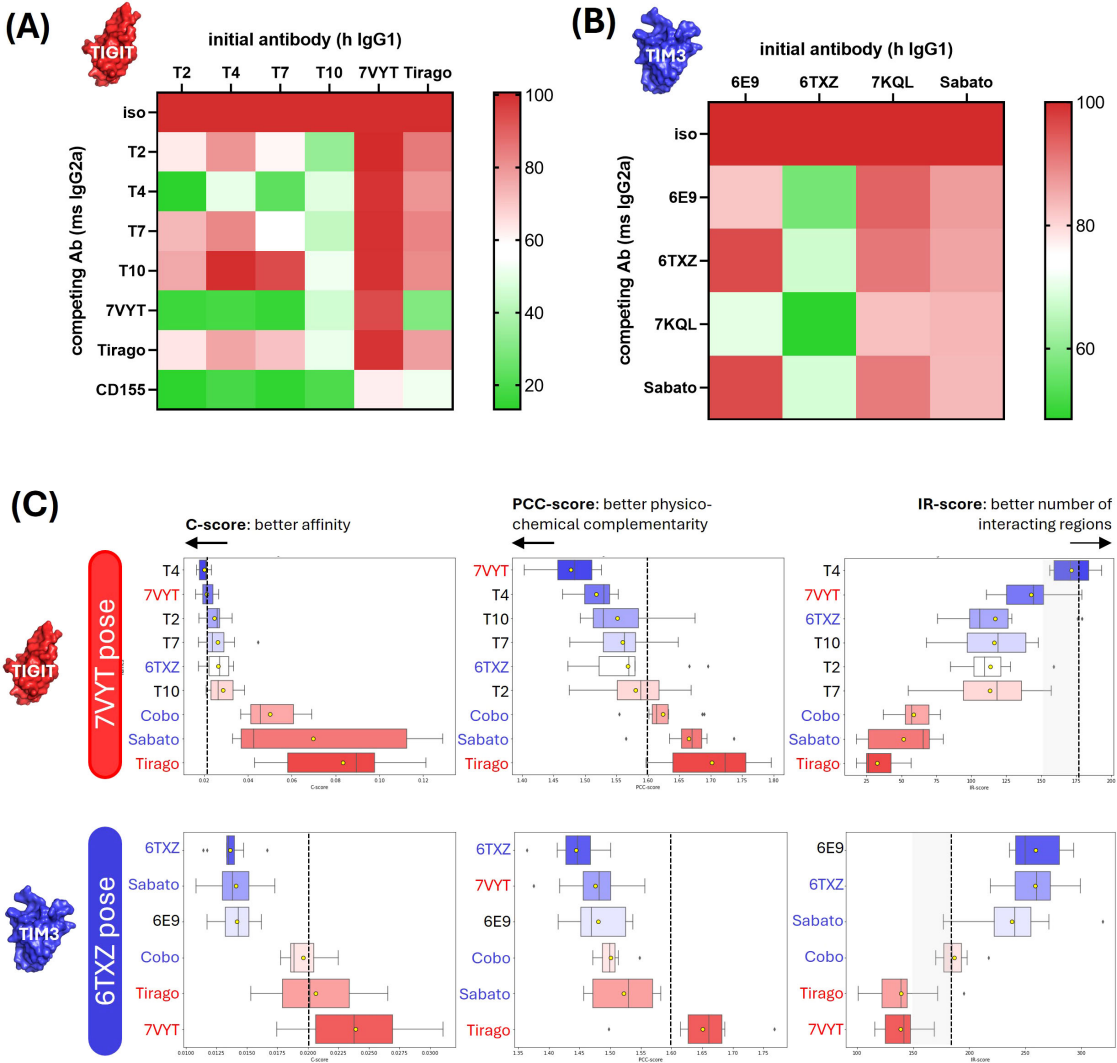
Affinity evaluation and structural model definition of the antibody-antigen complexes. **(A, B)** Experimental competition assays. Pairwise competition assays of TIGIT binders **(A)** or anti-TIM3 antibodies **(B)** were performed by flow cytometry. Briefly, the Fc fragments of our human IgG1 candidates were replaced by mouse IgG2a Fc fragments. HEK293 cells were transiently transfected with the Flag-tagged target gene and co-incubated the human IgG1-formatted and the mouse IgG2a-formatted candidates. In the case of TIGIT, the competition was also evaluated with its natural ligand CD155 fused to a mouse IgG2a Fc fragment. The target expression was monitored with a PE-coupled anti-Flag antibody and the binding of the human IgG1-formatted antibodies was followed with an APC-coupled antibody. The percentage of APC+PE+ cells (among the PE+ subset) was indicated as a color gradient. **(C)** Affinity predictions and structural model definition. The antibodies were docked on selected epitopes (indicated in the left boxes). The affinity of the obtained complexes was computed as the C-score (left column) which was a combination of PCC-score (middle column) and IR-score (right column). The upper panels show the affinity predicted for the anti-TIGIT candidates docked on 7VYT epitope. The middle and lower panels show the affinity predicted for the anti-TIM3 antibodies docked on 6TXZ and 7KQL epitope, respectively.

In order to gain an approximate idea of the antibodies binding regions, competition assays were performed with reference antibodies which epitopes were partially or totally deciphered, i.e. 7VYT and Tiragolumab for the anti-TIGIT antibodies, and 6TXZ, 7KQL and Sabatolimab for the anti-TIM3 antibodies (**Figures 7A, B**). Competitions were assessed by flow cytometry, as pairwise displacements of human IgG1 by mouse IgG2a, on cells overexpressing TIM3 or TIGIT. In the case of TIGIT, the displacement of the antibodies was also evaluated after addition of its ligand CD155 fused to a mouse IgG2a Fc fragment. All 4 anti-TIGIT hIgG1 were drastically displaced by 7VYT and CD155, and to a lesser extent by the Tiragolumab (**Figure 7A**). The 3 proteins share highly overlapping binding sites, located in the vicinity of the FG and CC’ loops (**Supplementary Figure S6**). The modeled structures of the 4 TIGIT candidates were docked and aligned onto 7VYT orientation on TIGIT, and the affinity evaluated according to our method (**Figure 7C**). The 7VYT itself was introduced in the procedure, as a positive control. The Tiragolumab, which binds roughly the same epitope as 7VYT but with a very different VH-VL orientation, as well as the anti-TIM3 6TXZ, Sabatolimab and Cobolimab, were introduced as negative controls. First, it can be observed that the 7VYT was highly ranked in the final C-score, proving the efficacy of the method to correctly evaluate affinity and to rank high the best orientation (**Figure 7C**, upper panels). Conversely, the Tiragolumab, was ranked last, hence showing that the orientation which dictates the facing residues and the subsequent binding of the two moieties was properly taken into account in our computations. As expected, the anti-TIM3 6TXZ, Cobolimab, and Sabatolimab were poorly evaluated for their binding to TIGIT. Among our 4 candidates, T4 ranked very high, above the reference 7VYT, allowing us to deduce that i/ it bound TIGIT in the imposed VH-VL orientation and that ii/ it displayed a very good affinity. T2, and to a lesser extent T7, ranked similarly to 7VYT, indicating that they likely share a similar orientation with 7VYT, though not perfectly overlapping. T10, which ranked closely to the negative control 6TXZ, probably displayed a different orientation.

6E9 was subjected to the same procedure. The competition assays showed that 6E9, 6TXZ and 7KQL have overlapping epitopes (**Figure 7B**). 6TXZ and 7KQL crystal structures show that their epitopes are highly overlapping, also located in the vicinity of the CC’-FG loops, and that their orientations on TIM3 are very similar (**Supplementary Figure S6**). 6TXZ was selected as the reference pose because it shares approximately the same paratope size as 6E9. 6TXZ and the Sabatolimab were introduced as positive controls in the computations. No crystal structure is disclosed for the Sabatolimab, but structural evidence published by (20) show that its epitope strongly overlaps with 6TXZ and that they share globally the same VH-VL orientation. The affinity evaluation on 6TXZ showed a satisfying ranking of the positive reference Sabatolimab and of 6TXZ itself (**Figure 7C** middle panels). As expected, the anti-TIGIT negative controls Tiragolumab and 7VYT ranked poorly on TIM3. 6E9 ranked high, showing that it shared 6TXZ pose and orientation on TIM3.

## 4 Discussion

When classically performed, antibody discovery requires numerous rounds of molecular (structure, PTMs…), physico-chemical (solubility at high concentrations, manufacturability at large scale…), and functional (efficacy, specificity, toxicity…) characterizations, as well as optimization steps (affinity maturation, humanization…) in order to qualify the candidate molecules before preclinical and clinical trials, and finally allow them to become therapeutics. Here, we demonstrate the added value of our AI-based tools in enhancing antibody lead characterization and facilitating more rational decision-making during the selection process.

In this work, 39 individual scFv candidates were selected *in vitro* by phage display and the detailed developability study showed for the 5 binding IgG candidates. They were subjected to several layers of computational analysis which revealed that they were globally equivalent and satisfying for most of the parameters, i.e. the sequence identity with patented Ab, the exposed PTM motifs, the CDR lengths and charges, the humanness or the predicted off-targets. Two parameters were discriminating. Analysis of their surface physico-chemical properties revealed that 6E9, T4, and to a lesser extent T7 exhibited good aggregation - solubility profiles, but that T2 and T10 exhibited intense hydrophobic regions that could drive them to aggregation, probably hampering their future in terms of high scale production, storage and their commercial development. Antibodies administration routes (intravenous, subcutaneous, intramuscular, and intraperitoneal) limit injection volumes and require very high concentrations to be achieved in the pharmaceutical preparations (typically 50 to 200 mg/ml). This is particularly challenging for aggregation and often unsolvable by adapting the formulation if the antibody is not good at the beginning. Because the method is fast at identifying the surface patches and computing the aggregation scores, large collections of antibodies can be evaluated simultaneously. Moreover, in silico-generated mutational variants of one antibody could also be evaluated swiftly, if rectifying the antibody’s properties is expected. The second discriminating criterion was the affinity-based validation of the structural models of the antibody-antigen complex. Obtaining such a model is a huge added value for the future development of the antibody. Not only it allows to obtain a precise definition of the epitope and to secure the IP protection, but also to provide solid bases for functional studies and optimization experiments such as affinity maturation or interspecies binding study to prelude preclinical trials. Reliable structural models were obtained for T4 and 6E9, ultimately ranking them as the best candidates against TIGIT and TIM3 respectively.

Three decades ago, Lipinski’s proposed his “rule of 5” to predict the drug-likeliness of small chemical compounds from their physicochemical properties (43). While Lipinski’s rules are not applicable to the size and complexity of biologics, an analogous tool would be highly valuable. Among the 39 initial candidates, 33 displayed complete VH and VL sequences and only 5 maintained a binding activity at the end of the biological process once reformatted as IgGs. In order to gain insight into this attrition rate, we subjected *a posteriori* the 33 candidates to the physicochemical tools of our developability analysis i.e. aggregation score evaluation, CDR analysis (length and charge), PTM motifs identification and their exposure in the CDR, and the affinity evaluation as computed by our PC, PCC and IR scores. Data are summarized in the table shown in **Supplementary Figure S7A**. Very interestingly, only a very few leads showed no warnings in any of the dimensions studied: the references 7KQL, 6TXZ and 7VYT, the 2 successful candidates 6E9 and T4 and one false positive: 3F8. This latter one was a very weak binder in the scFv screening, and not a binder once reformatted in IgG. The a posteriori analysis revealed that its aggregation score flirted with the threshold, probably highlighting some stability issue. Whereas these results are highly encouraging in showing that our tools are successful in identifying the good leads when used in combination, it cannot be excluded that other dimensions might be interesting to investigate, like the melting temperature (Tm) for example. Importantly, when comparing the sequence identities of all the antibodies (the candidates isolated during the phage display, the top leads validated computationally and experimentally and the reference antibodies), no similarity cluster could be observed (**Supplementary Figure S7B**). This wide sequence diversity ranging from 48.5 to 96.1% at the full-length level, and from 20.4 to 89.3% when considering only the 6 CDRs shows unequivocally that our method is effective at comparing evolutionary distant antibodies, and that it does not favor antibodies that are close to existing references.

Affinity is the central parameter of antibody discovery: the initial selection of antibody hits is performed on affinity criteria, and affinity is the prime parameter evaluated when the antibody is modified along its development. Mastering affinity prediction holds the hope to be able to control two pivotal aspects of antibody discovery: i/being able to find within a very large collection of antibodies the ones binding a desired target, which means doing computational antibody selection, and ii/being able to predict the affinity of antibody variants, which means doing computational antibody maturation. We showed here the efficacy of our structure-based affinity prediction tool which computes parameters of the antibody-antigen complex interface. Other methods based on the same principle of structure study have been published, the most efficient being RosettaAntibodyDesign (44, 45). Unlike large language models-based tools (46–48), our method does not require training on biological data generated specifically for the studied complex. Being target agnostic, it can rapidly be applied to new targets (or variants of the same target through modeling). The availability of reference antibodies is not strictly mandatory, yet it helps providing an additional cross-validation layer of the predictions. The amount of sequential and structural data available allows to consider achievable the reliable modeling of large collections of conventional IgGs. However, atypical antibody formats (e.g. bispecific antibodies, nanobodies) or conventional antibodies displaying extra-long CDRs for example may challenge the models. While only a limited number of antibodies were considered in this study, our affinity method proved effective at ranking both positive and negative binders on selected epitopes, setting the stage for expanding the pool of initial molecules investigated, potentially reaching full NGS repertoires in the future. Coupled with developability evaluation to eliminate at the outset the leads displaying unsuitable properties, the foundations for an intelligent antibody selection method are now in place.

TIGIT (VSIG9) and TIM3 (HAVCR2/CD366) both belong to the Ig-like/V-set structural family of proteins. As such, and despite their very low sequence identity (20%), TIM3 and TIGIT share a high structural homology (RMSD = 5.59 Å). However, they are structurally very different in their ligand-binding domains. Within the TIM family, four highly conserved cysteines (C58, C63 in the CC’ loop, C52 in the C strand, and C110 in the F strand in TIM3) form disulfide bonds that constrain the CC’ and FG loops in a closed conformation unique among the V-set family (49). The aim of this study was to identify antagonist antibodies inhibiting the binding of TIM3 or TIGIT to their respective partners. TIM3 has 3 functional partners, *i.e.* CEACAM1, Galectin 9 and phosphatidylserine. Co-crystal structures of TIM3 and either CECAM1 (PDB 4QYC, (50)) or PtdSer (PDB 3KAA, (51)) show they both bind in or around the region delimitated by the FG and CC’ loops. Gal9 binding site, on the other hand, may involve distant residues and glycosylated chains interactions and remains to fully explore (52–54). TIGIT binding to its partners CD155 (PVR, PDB 3UDW (55)) and Nectin-2 (PDB 5V52 (56)) also occurs on the region delimited by the FG and CC’ loops. Consistently with the antagonistic effect expected of ICI, publicly disclosed antibodies raised against TIM3 or TIGIT are mostly located in the same region. Such is the case for the Tiragolumab, Sabatolimab, 7VYT/MG113, 6TXZ/M6903, 7KQL/Tim3.18 used as competitors in wet-lab experiments and as references during the computations. All results converged at locating the 5 newly discovered antibodies on the ligands binding sites of TIM3 and TIGIT, allowing to anticipate the expected antagonistic effect. Functional assays are now necessary to investigate the antibodies’ efficacy on T cells exhaustion reversal. The expression of T-cell activation markers (CD69, CD25) and the inhibition of cytokine release (e.g., IFN-γ, IL-2, TNF-α) will be investigated in priority.

Immune checkpoints constitute a vast family of inhibitory and activating receptors. They belong to a dynamic system that responds to the TME variations. Their expressions on cancer cells were reported to vary along cancer development (for example, (57, 58)), but also subsequently to treatments used in patients. Changes in the expression of PD-1, PD-L1 and CTLA-4, among others, were observed after chemotherapy, radiotherapy, or a combination of them (59). Anti-PD-1 treatment itself was also shown to alter the RNAseq profile of tumors (60). If we consider the tumor as an evolutive system, precise phenotyping should be a prerequisite before starting any ICI therapies. A toolbox of ICI ready to be combined would allow to tackle cancer with personalized medicine strategies.

## Data Availability

The datasets presented in this article are not readily available because generated data are antibody sequences which remain protected by the secret. Requests to access the datasets should be directed to astrid.musnier@mabsilico.com.
