# Supplementary material for: AI-enhanced profiling of phage-display-identified anti-TIM3 and anti-TIGIT novel antibodies
Source: Front Immunol. 2025 Mar 11;16:1499810. doi: 10.3389/fimmu.2025.1499810 (PMC11933058; doi:10.3389/fimmu.2025.1499810)
Supplement: Supplementary file 1 [file DataSheet1.pdf]

Supplementary Material

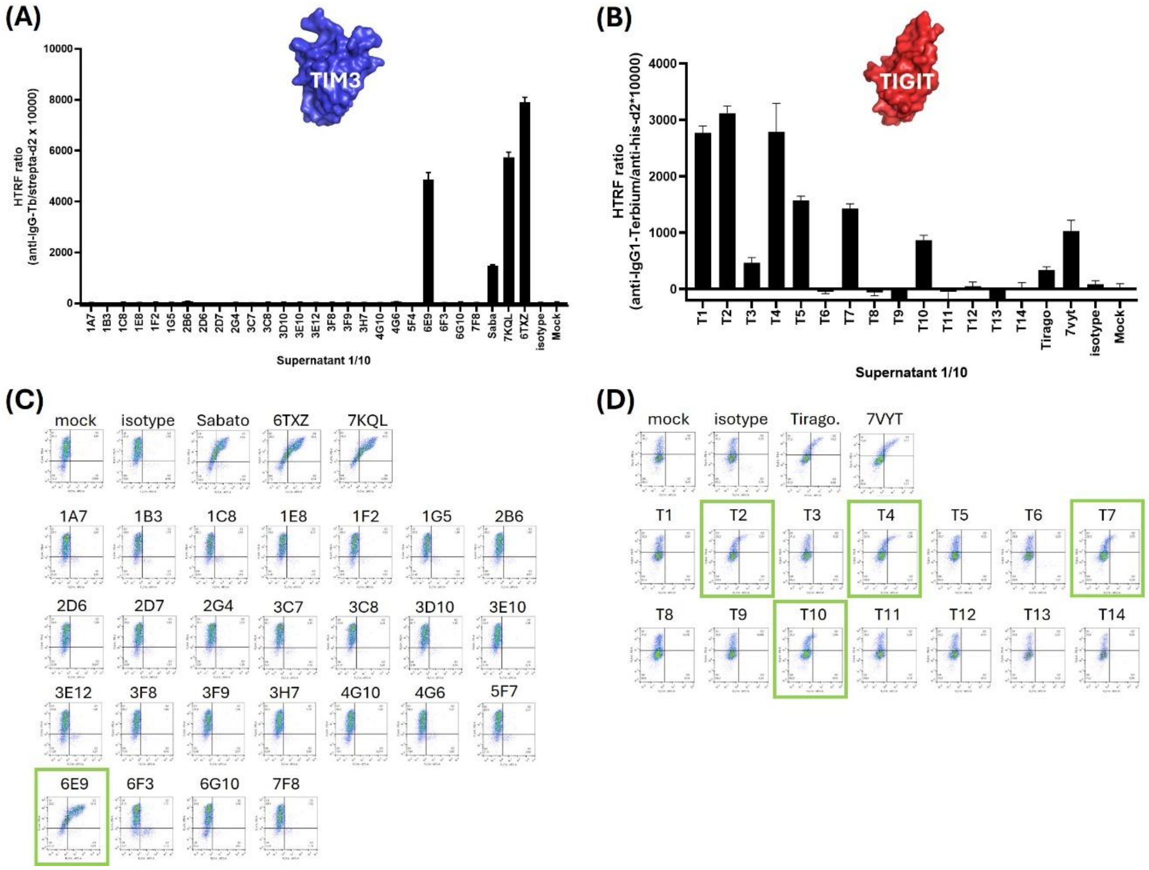

Supplementary Figure 1. Binding validation of the IgG1-reformatted scFv identified during phage display screening campaign. The scFv identified by ELISA during the phage display campaign were reformatted as human IgG1 and produced in HEK293 supernatant. **(A) and (B)** HTRF-based binding validation. The recombinant extracellular domains of TIM3 **(A)** or TIGIT **(B)** fused to a biotinylated Avitag, were incubated with the antibodies (supernatant diluted to 1/10). Antibodies and targets were detected with fluorophore-coupled sensors: d2 acceptor coupled to an anti-IgG and terbium donor coupled to the streptavidin. The binding was assessed as the energy transfer between the donor and acceptor and computed as the HTRF ratio: 665nm acceptor emission/ 620 nm donor emission x 10,000. Curves were fitted mathematically with GraphPad Prism software. **(C) and (D)** Cytometry-based binding validations. HEK293 cells were transiently transfected with the Flag-tagged full-length TIM3 **(C)** or TIGIT **(D)** and incubated with antibodies. The target expression was monitored with a PE-coupled anti-Flag antibody (signal plotted on the y axis), and the binding of the antibodies was followed with an APC-coupled anti-IgG (signal plotted on the x axis). Sabatolimab, 6TXZ, 7KQL, Tiragolumab and 7VYT were used as positive references, and a human anti-IgG1 isotype as a negative control. Green frames highlight binder candidates.

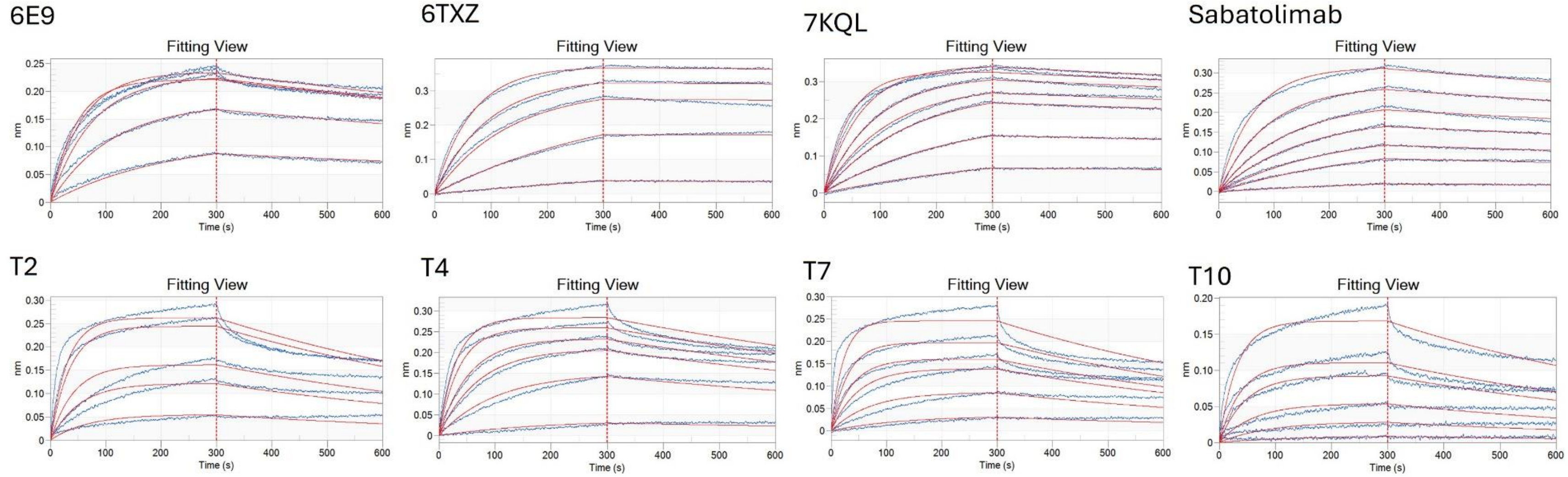

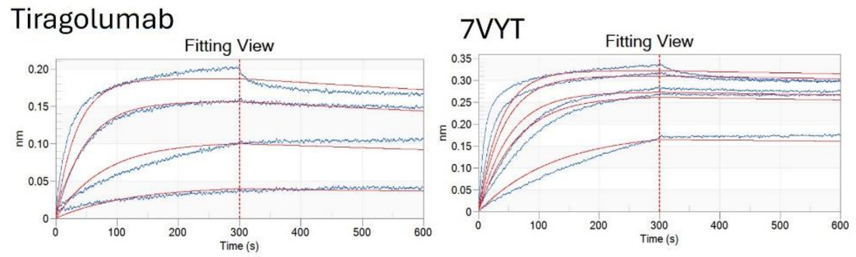

	KD (M)	KD Error	kon(1/Ms)	kon Error	kdis(1/s)	kdis Error	Full X^2	Full R^2	RMax
**6E9**	**1.30E-09**	1.90E-11	4.29E+05	2.41E+03	5.56E-04	7.52E-06	0.1101	0.9905	0.2272
**7KQL**	**1.01E-09**	1.55E-11	2.16E+05	5.68E+02	2.19E-04	3.29E-06	0.0785	0.9982	0.3294
**6TXZ**	**2.00E-10**	3.42E-11	1.53E+05	6.48E+02	3.06E-05	5.23E-06	0.1061	0.9977	0.3717
**Sabatolimab**	**2.44E-09**	3.09E-11	1.57E+05	5.78E+02	3.82E-04	4.63E-06	0.0775	0.9978	0.3212
**T2**	**3.19E-09**	6.25E-11	4.50E+05	6.36E+03	1.44E-03	1.95E-05	0.8171	0.9729	0.2739
**T4**	**2.00E-09**	3.03E-11	4.48E+05	3.79E+03	8.96E-04	1.13E-05	0.4085	0.9845	0.2927
**T7**	**2.53E-09**	5.19E-11	6.37E+05	1.01E+04	1.61E-03	2.11E-05	0.6927	0.959	0.2538
**T10**	**3.07E-09**	5.06E-11	4.96E+05	6.00E+03	1.52E-03	1.70E-05	0.1571	0.9816	0.1771
**Tiragolumab**	**6.81E-10**	3.43E-11	4.10E+05	4.43E+03	2.79E-04	1.37E-05	0.1307	0.984	0.1892
**7VYT**	**1.89E-10**	3.37E-11	3.93E+05	4.10E+03	7.43E-05	1.32E-05	0.8187	0.9553	0.3227

Supplementary figure S2: Complete BLI data. Human IgG1 of the 5 final candidates and reference antibodies were produced in HEK293 supernatant, concentrated and dosed. BLI AHC biosensors were loaded with the candidate antibodies. The association with the target was measured on 3 to 6 concentrations of TIM3 of TIGIT, and the dissociation was passively induced in PBS. K_D and other binding kinetic values indicated in the table were obtained from the simultaneous fit of all the shown curves. The binding kinetics (kon, kdis) and affinity constant (KD) for the 5 final candidate antibodies and their references were summarized in the table.

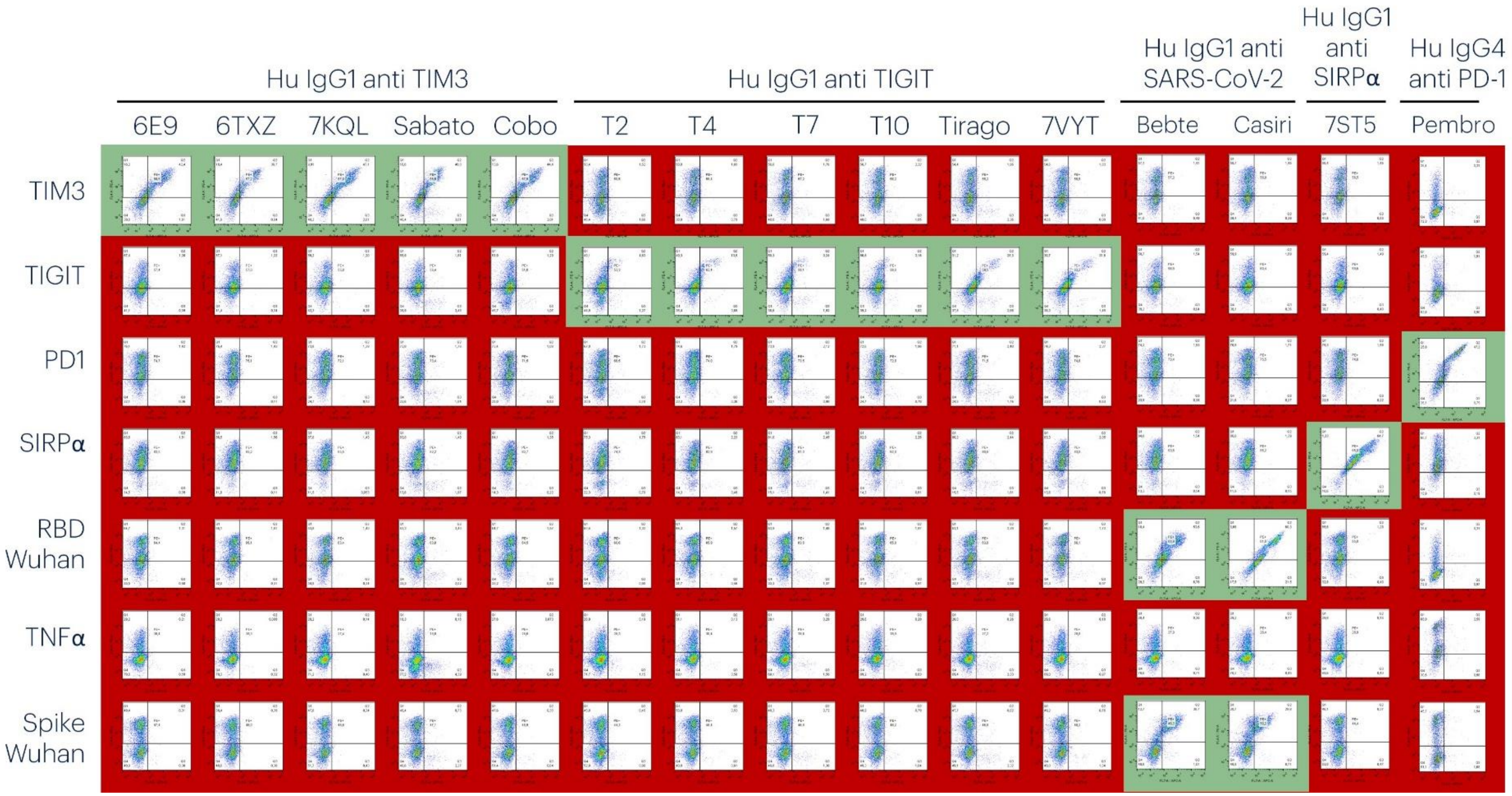

Supplementary figure S3: Specificity assessment cytometry plots. HEK293 cells were transiently transfected with either one of the Flag-tagged full-length targets (TIM3, TIGIT, PD1, SIRP-α, TNFα, SARS-CoV-2 Spike or its RBD domain) anchored to the membrane via the CD8 transmembrane domain. The target expression was monitored with a PE-coupled anti-Flag antibody (signal plotted on the y axis), and the binding of the antibodies was followed with an APC-coupled anti-IgG (signal plotted on the x axis). Binding was evaluated as the apparition of a APC+PE+ population (top right quartile). Red frames indicate plots where no binding was observed between target and antibody while green frames designate plots where antibody binds to the target on HEK293 cells.

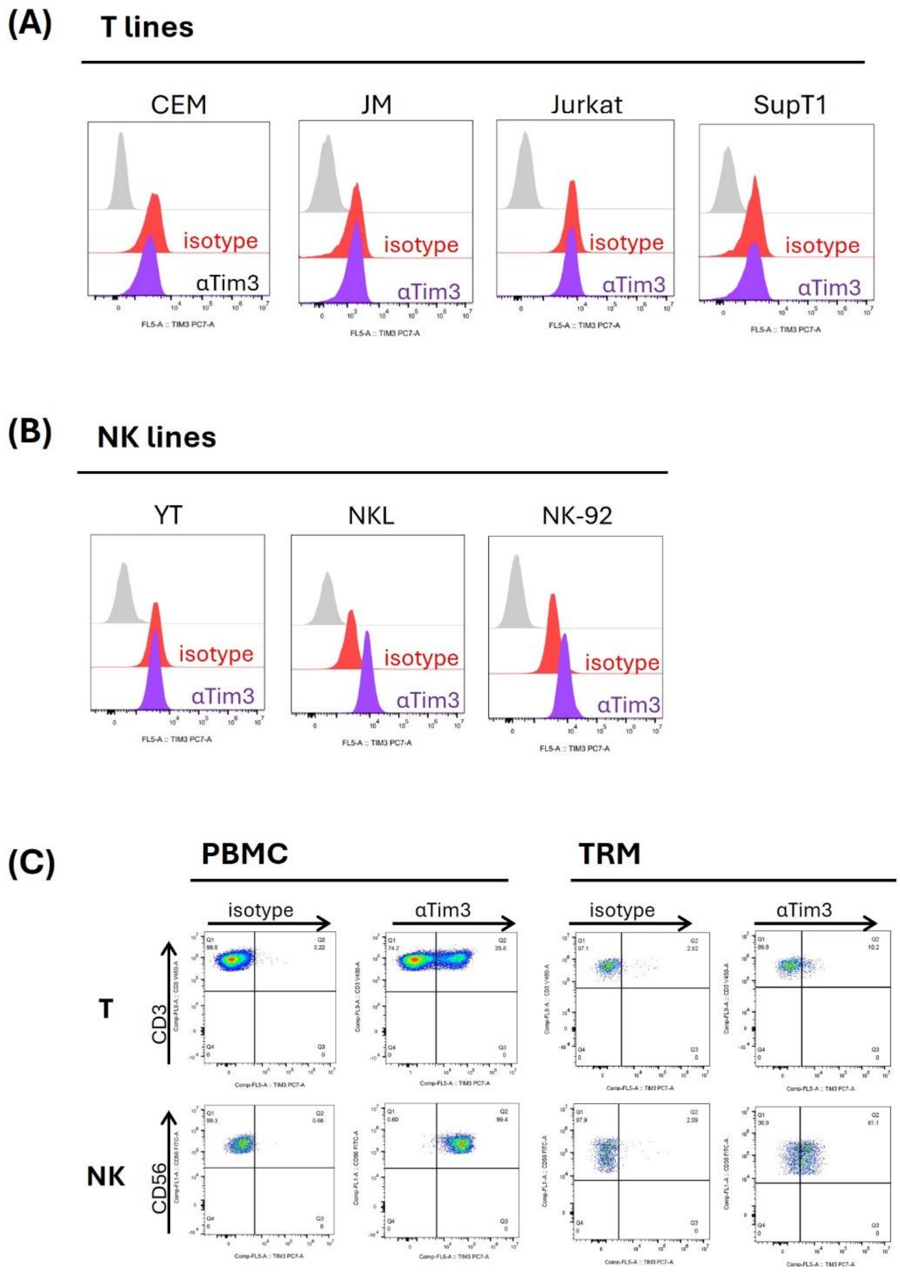

Supplementary figure S4: TIM3 expression on immune-relevant cells. The detection of endogenous TIM3 was performed by flow cytometry with commercial antibody or isotype control. Four T cells lines (CEM, JM, Jurkat and SupT1) **(A)**, three NK cells lines (YT, NKL and NK-92) **(B)** and primary activated PBMC and TRM cultivated with TGFβ and IL15 **(C)** were investigated. The T and NK subpopulation of the PBMC and TRM were optically isolated by staining CD3 and CD56 (CD3+ T Lymphocytes and CD3- CD56+ NK Lymphocytes).

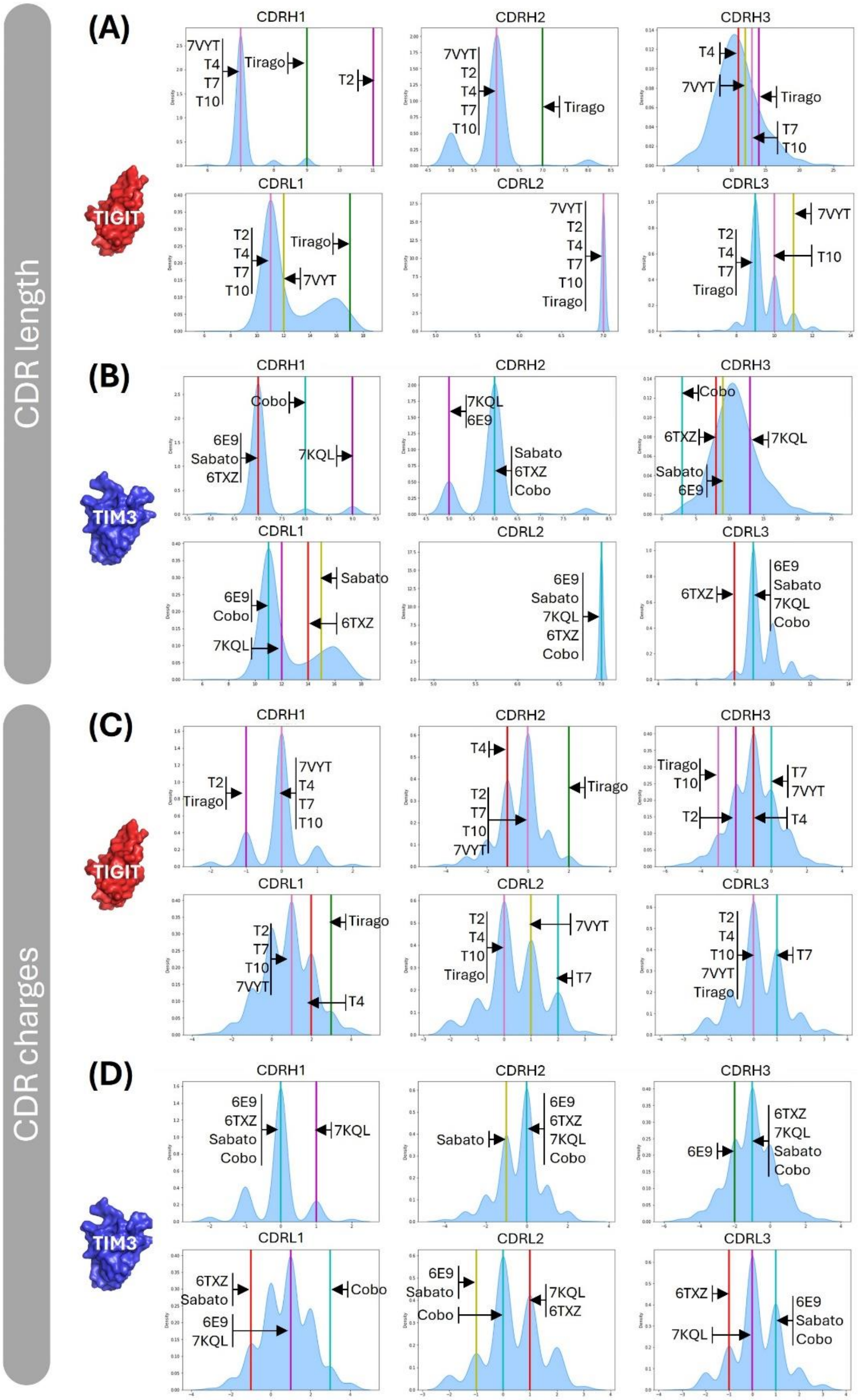

Supplementary figure S5: CDR lengths and charges analysis. The CDR lengths **(A, B)** and charges **(C, D)** were computed for 735 INN antibodies and their distribution showed in blue. Our anti-TIGIT **(A, C)** and anti-TIM3 **(B, D)** candidates as well as their references were compared with the INN distribution.

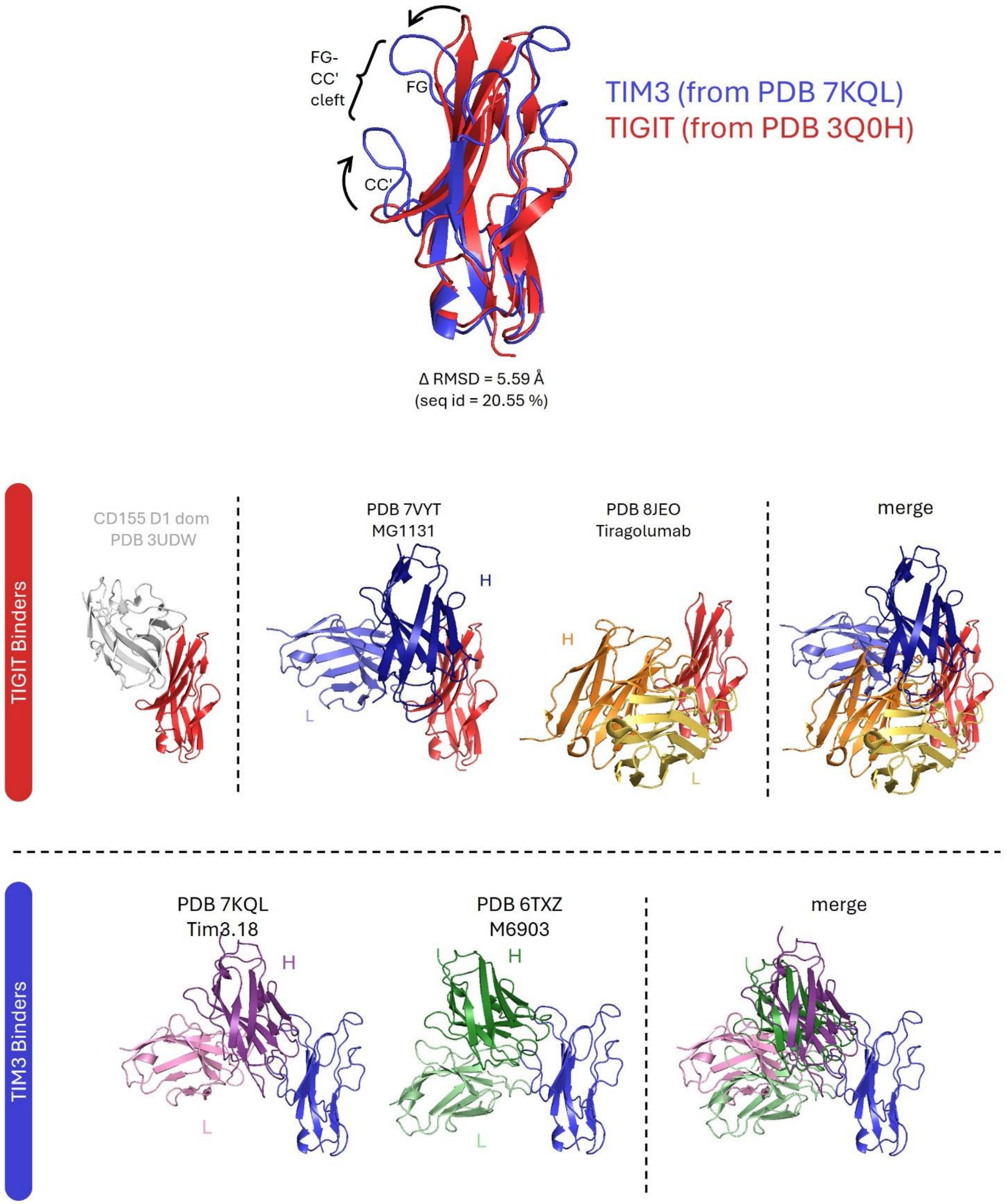

Supplementary figure S6: Visual representation of publicly disclosed anti-TIGIT and anti-TIM3 antibodies structures. The 3D structure overlay of TIGIT (from PDB 3Q0H) and TIM3 (from pdb 7KQL) were represented in red and blue. The FG-CC' cleft is indicated. The 3D structure of CD155 (pdb 3UDW), MG113 (pdb 7VYT), and Tiragolumab (pdb 8JEO) were represented with TIGIT individually or merged together. The 3D structures of Tim3.18 (pdb 7KQL) and M6903 (pdb 6TXZ) were represented with TIM3 individually or merged together.

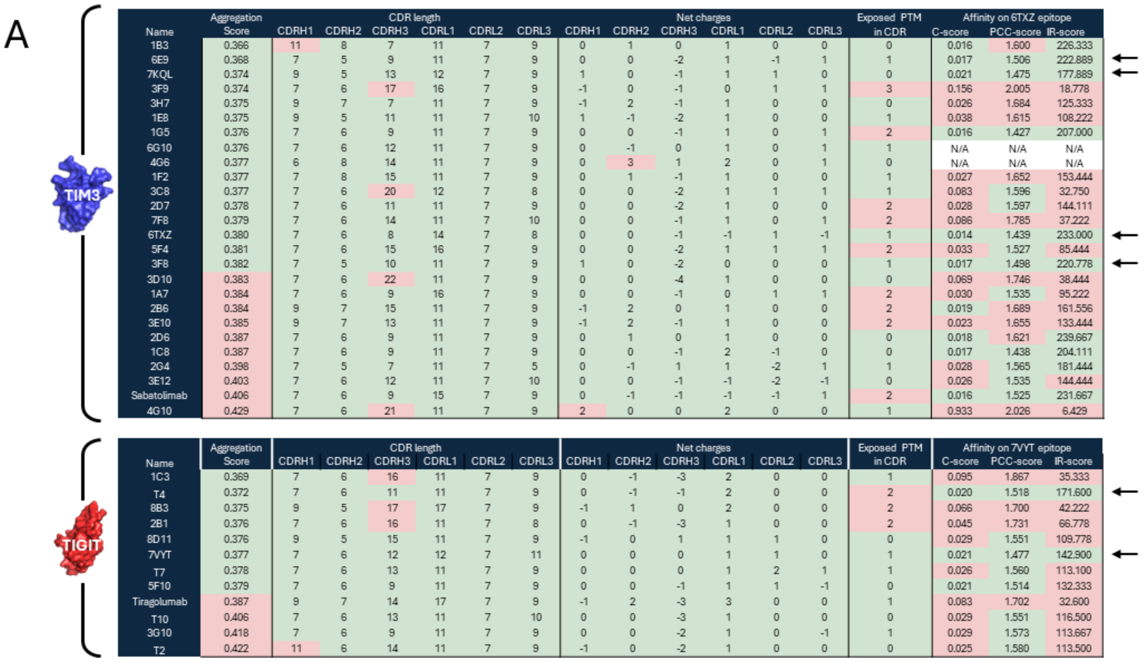

Name	Aggregation Score	CDR length CDRH1	CDRH2	CDRH3	CDRL1	CDRL2	CDRL3	Net charges CDRH1	CDRH2	CDRH3	CDRL1	CDRL2	CDRL3	Exposed PTM in CDR	Affinity on 6TXZ epitope C-score	PCC-score	IR-score
1B3	0.366	11	8	7	11	7	9	0	1	0	1	0	0	0	0.016	1.600	226.333
6E9	0.368	7	5	9	11	7	9	0	0	-2	1	-1	1	1	0.017	1.506	222.889
7KQL	0.374	9	5	13	12	7	9	1	0	-1	1	1	0	0	0.021	1.475	177.889
3F9	0.374	7	6	17	16	7	9	-1	0	-1	0	1	1	3	0.156	2.005	18.778
3H7	0.375	9	7	7	11	7	9	-1	2	-1	1	0	0	0	0.026	1.684	125.333
1E8	0.375	9	5	11	11	7	10	1	-1	-2	1	0	0	1	0.038	1.615	108.222
1G5	0.376	7	6	9	11	7	9	0	0	-1	1	0	1	2	0.016	1.427	207.000
6G10	0.376	7	6	12	11	7	9	0	-1	0	1	0	1	1	N/A	N/A	N/A
4G6	0.377	6	8	14	11	7	9	0	3	1	2	0	1	0	N/A	N/A	N/A
1F2	0.377	7	8	15	11	7	9	0	1	-1	1	0	0	1	0.027	1.652	153.444
3C8	0.377	7	6	20	12	7	8	0	0	-2	1	1	1	1	0.083	1.596	32.750
2D7	0.378	7	6	11	11	7	9	0	0	-2	1	0	0	2	0.028	1.597	144.111
7F8	0.379	7	6	14	11	7	10	0	0	-1	1	0	1	2	0.086	1.785	37.222
6TXZ	0.380	7	6	8	14	7	8	0	0	-1	-1	1	-1	1	0.014	1.439	233.000
5F4	0.381	7	6	15	16	7	9	0	0	-2	1	1	1	2	0.033	1.527	85.444
3F8	0.382	7	5	10	11	7	9	1	0	-2	0	0	0	1	0.017	1.498	220.778
3D10	0.383	7	6	22	11	7	9	0	0	-4	1	0	0	0	0.069	1.746	38.444
1A7	0.384	7	6	9	16	7	9	0	0	-1	0	1	1	2	0.030	1.535	95.222
2B6	0.384	9	7	15	11	7	9	-1	2	0	1	0	0	2	0.019	1.689	161.556
3E10	0.385	9	7	13	11	7	9	-1	2	-1	1	0	0	2	0.023	1.655	133.444
2D6	0.387	7	6	9	11	7	9	0	1	0	1	0	0	0	0.018	1.621	239.667
1C8	0.387	7	6	9	11	7	9	0	0	-1	2	-1	0	0	0.017	1.438	204.111
2G4	0.398	7	5	7	11	7	5	0	-1	1	1	-2	1	1	0.028	1.565	181.444
3E12	0.403	7	6	12	11	7	10	0	0	-1	-1	-2	-1	0	0.026	1.535	144.444
Sabatolimab	0.406	7	6	9	15	7	9	0	-1	-1	-1	-1	1	2	0.016	1.525	231.667
4G10	0.429	7	6	21	11	7	9	2	0	0	2	0	0	1	0.933	2.026	6.429

Name	Aggregation Score	CDR length CDRH1	CDRH2	CDRH3	CDRL1	CDRL2	CDRL3	Net charges CDRH1	CDRH2	CDRH3	CDRL1	CDRL2	CDRL3	Exposed PTM in CDR	Affinity on 7VYT epitope C-score	PCC-score	IR-score
1C3	0.369	7	6	16	11	7	9	0	-1	-3	2	0	0	1	0.095	1.867	35.333
T4	0.372	7	6	11	11	7	9	0	-1	-1	2	0	0	2	0.020	1.518	171.600
8B3	0.375	9	5	17	17	7	9	-1	1	0	2	0	0	2	0.066	1.700	42.222
2B1	0.376	7	6	16	11	7	8	0	-1	-3	1	0	0	2	0.045	1.731	66.778
8D11	0.376	9	5	15	11	7	9	-1	0	1	1	1	0	0	0.029	1.551	109.778
7VYT	0.377	7	6	12	12	7	11	0	0	0	1	1	0	1	0.021	1.477	142.900
T7	0.378	7	6	13	11	7	9	0	0	0	1	2	1	1	0.026	1.560	113.100
5F10	0.379	7	6	9	11	7	9	0	0	-1	1	1	-1	0	0.021	1.514	132.333
Tiragolumab	0.387	9	7	14	17	7	9	-1	2	-3	3	0	0	1	0.083	1.702	32.600
T10	0.406	7	6	13	11	7	10	0	0	-3	1	0	0	0	0.029	1.551	116.500
3G10	0.418	7	6	9	11	7	9	0	0	-2	1	0	-1	1	0.029	1.573	113.667
T2	0.422	11	6	14	11	7	9	-1	0	-2	1	0	0	0	0.025	1.580	113.500

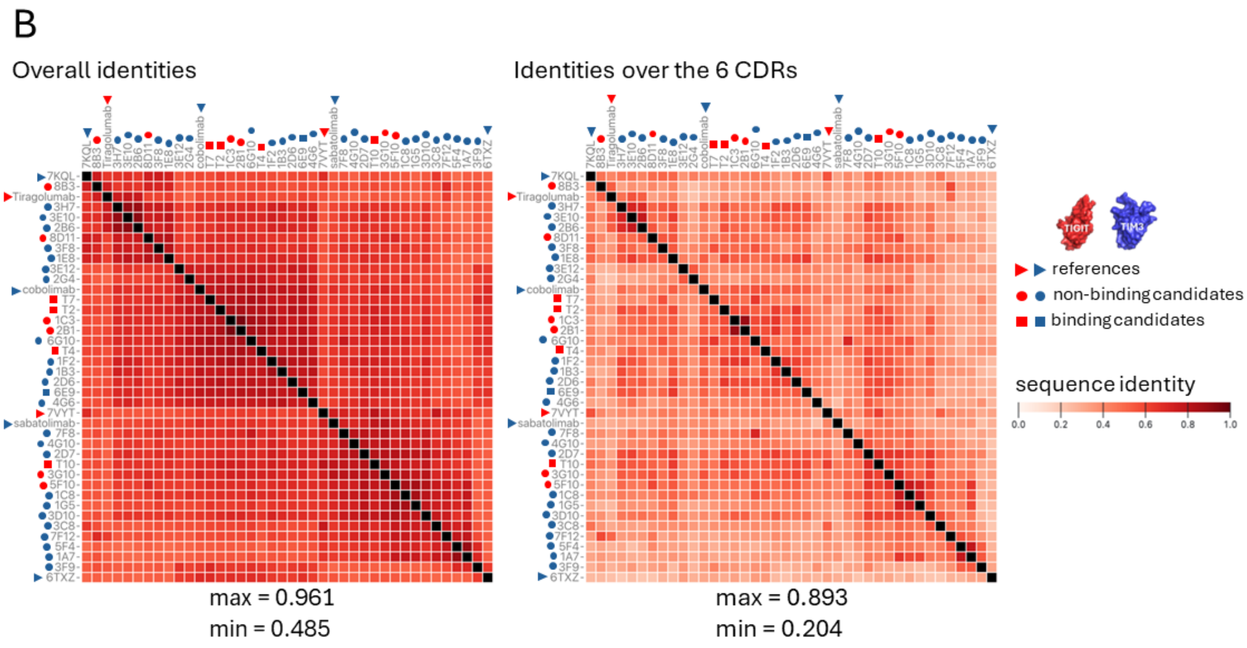

Supplementary figure S7: Multidimensional analysis of all the candidates identified by phage display. (A) Developability and affinity parameters were evaluated for all the anti-TIM3 (upper table) and anti-TIGIT (lower table). Developability analysis includes the predicted aggregation scores, the CDRs lengths and charges analysis, and the number of exposed PTMs motifs in the CDRs. The affinity scores were predicted on 6TXZ epitope on TIM3 or 7VYT epitope on TIGIT, for all but 2 antibodies which revealed aberrant scores (N/A). The values above the threshold are shown in green, the ones below in pink. The arrows show the 6 antibodies exhibited values above the threshold in all the dimensions studied. **(B)** Pairwise sequence identities of the antibodies for their full-length VH and VL sequences (left pannel), and their 6CDRs (right pannels).
